# Mid- and late-life cardiovascular health indicators and changes in biological ageing Markers; A multi-cohort study

**DOI:** 10.1016/j.ebiom.2025.106016

**Published:** 2025-11-11

**Authors:** Nigus Gebremedhin Asefa, Yi-Han Hu, Zhiguang Li, Yinan Zheng, Osorio Meirelles, Jorge Martinez Romero, Donald M. Lloyd–Jones, Pei-Lun Kuo, Valborg Gudmundsdottir, Toshiko Tanaka, Thor Aspelund, Lifang Hou, Vilmundur Gudnason, Luigi Ferrucci, Lenore J. Launer

**Affiliations:** a Laboratory of Epidemiology and Population Sciences, National Institute on Aging, Baltimore, MD, USA; b Division of Cancer Epidemiology and Prevention, Department of Preventive Medicine, Northwestern University Feinberg School of Medicine, Chicago, IL, USA; c Robert J. Havey MD Institute for Global Health, Northwestern University Feinberg School of Medicine, Chicago, IL, USA; d Translational Gerontology Branch, National Institute on Aging, Baltimore, MD, USA; e Icelandic Heart Association, Kopavogur, Iceland; f Faculty of Medicine, University of Iceland, Reykjavik, Iceland

**Keywords:** Biological ageing, Epigenetic ageing, DNA methylation, DunedinPACE, Ageing, Lifestyle, Cardiovascular health

## Abstract

**Background:**

Cardiovascular (CV) health-related risk factors may influence the epigenetic-based pace of biological ageing (BA). However, given that early-life lifestyle factors can have lasting effects on DNA methylation (DNAm), observed associations may reflect cumulative exposures rather than short-term changes in older adults. We investigated whether CV risk factors are associated with changes in the pace of ageing in midlife and older adults.

**Methods:**

We analysed baseline DNA methylation data from 4848 participants across three cohorts, AGES-RS (n = 2602), CARDIA (n = 1568), and InCHIANTI (n = 678), using Illumina arrays, with two to four time points per cohort. Pace of ageing was measured using DunedinPACE (DDPACE). Cardiovascular risk factors included smoking status, DNAm-derived pack-years, physical activity (PA), body mass index (BMI), systolic and diastolic blood pressure (SBP and DBP), total cholesterol, blood fasting glucose, and their composite score (adapted Life's Simple 7 [adapted-LS7]). We conducted three analyses: (1) prospective analysis of CV risk factors (exposures) and DDPACE (outcome); (2) delta analysis of changes in DDPACE; and (3) shift analysis focusing on participants whose pace of ageing accelerated (accelerators: ≥1 SD above the mean) or decelerated (decelerators: ≤1 SD) over time. After excluding individuals with consistently average, fast, or slow ageing patterns, we conducted each analysis at the 5-year and 9^+^-year follow-ups.

**Findings:**

Across cohorts, >55% were female. Mean (SD) age ranged from 40.3 (3.6) in CARDIA to 76.3 (5.2) in AGES-RS. DDPACE also varied: 0.92 (0.13) in CARDIA vs. 1.10 (0.11) in AGES-RS. Within 5-year follow-up, smoking (current and former), pack-years of smoking, BMI, and blood glucose level were longitudinally associated with faster ageing (higher DDPACE; P < 0.05 (two-sided, linear mixed modeling)) in each cohort and in the meta-analyses. PA, diet, and higher adapted-LS7 scores were linked to slower ageing. Delta analyses confirmed consistent associations in AGES-RS, CARDIA, and the meta-analysis. In the meta-analysis of the 5-year shift model, higher pack-years, BMI, SBP, and DBP increased odds of being in the “accelerator” group compared to a “decelerator” (P < 0.05 (two-sided, logistic regression)), while higher adapted-LS7 scores increased odds of being a “decelerator” (P < 0.05 (two-sided, logistic regression)). Midlife prospective analysis in AGES-RS (∼age 50) showed similar patterns. Longer-term follow-ups (9+ years) in CARDIA and InCHIANTI showed similar but less consistent findings with the 5-year follow-up.

**Interpretation:**

Targeting modifiable CV risk factors, particularly smoking, physical inactivity, and poor cardiometabolic health, may help slow ageing and reduce age-related disease burden in midlife and older adults.

**Funding:**

National Institute on Aging, National Heart, Lung, and Blood Institute, Icelandic Heart Association, and Italian Ministry of Health.

**Research in context**

**Evidence before this study**

Our literature search in PubMed identified studies reporting associations between CV health-related factors, such as smoking cessation, physical activity, diet, and composite CV health scores, and epigenetic measures of biological ageing (BA).

However, evidence of different patterns of BA in humans is limited, as is the durability of the effect of a healthy life-style behaviour on BA.

Furthermore, the studies have focused on average associations, rather than on individuals who exhibit significant shifts (i.e., deterioration or improvement) in their BA trajectories.

Given that early-life lifestyle factors, like smoking, have long-lasting effects on DNA methylation, these observed associations may reflect cumulative exposures rather than dynamic changes in ageing during mid- or late-life.

Unlike previous studies, this study focused on individuals who exhibited shifts toward either accelerated or decelerated ageing, as measured by the DunedinPACE score, and investigated the association between CV health-related risk factors and these shifts.

**Added value of this study**

We demonstrate that CV-related factors, including smoking, physical activity, BMI, and adapted-LS7 scores, are longitudinally associated with the pace of ageing, even among midlife and older adults who experienced substantial shifts in their biological ageing trajectories.

**Implications of all the available evidence**

These findings suggest that interventions targeting lifestyle and behavioural factors in mid- and late-life, as captured by the adapted-LS7 score, may positively influence not only CV health but also broader ageing-related health outcomes.

## Introduction

Biological ageing (BA) reflects heterogeneity in cellular and molecular ageing processes.^1^^,^^2^ Advances in science have identified biomarker clocks, proxies for BA, that correlate with chronological age in almost every tissue.^3^ These clocks use percent DNA methylation data to estimate epigenetic age, predicting health outcomes such as frailty and mortality. Newer epigenetic clocks, trained on health indicators rather than chronological age alone, better predict mortality, frailty, and cardiometabolic risk.^2^^,^^4^^,^^5^ It has been shown that part of the variance in the epigenetic clocks is under genetic control but most importantly, can also be modified by environmental exposure and has been shown to change together with age-related changes in cells and tissues. Generally, genetically driven methylation changes occur until adulthood, then stabilize.^6^

Emerging evidence suggests BA does not necessarily follow the linear trajectory of chronological age. Studies suggest that healthy behaviours such as smoking, physical activity (PA), diet, and composite CV health scores can also change epigenetic-based BA measures.^5^^,^^7^ Some studies also suggest that BA clocks can be ‘reversed’ in animals and in humans, particularly when acute events resolve,^8^^,^^9^ or in short-term trials of healthier lifestyle interventions.^8^^,^^10^

However, evidence of different patterns of BA in humans is limited, as is the durability of the effect of a healthy life-style behaviour on BA. Furthermore, existing studies primarily examined associations between epigenetic ageing and these factors, without specifically focusing on individuals who exhibited substantial changes in their BA. Lifestyle factors such as smoking have well-documented, lasting effects on DNA methylation (DNAm),^11^ suggesting that observed associations may reflect cumulative, long-term influences rather than dynamic changes in BA in older adults. This study takes a different approach by focusing on individuals who demonstrated shifts toward either accelerated or decelerated ageing, as measured by the DunedinPACE (DDPACE) score.^1^ Specifically, we aimed to examine whether individual and combined CV risk factors contribute to these shifts, addressing a gap in understanding their relationship with BA in older adults. We hypothesized that, after excluding individuals who remained consistently on a fast or slow pace of ageing during follow-up, those transitioning toward acceleration would exhibit poorer CV health indicators compared with those shifting toward deceleration.

## Methods

### Study population

Data are from three longitudinal community-based samples, the Age, Gene/Environment Susceptibility-Reykjavik Study (AGES-RS), InCHIANTI and the Coronary Artery Disease in Young Adults (CARDIA). Description of these cohorts is provided in Supplementary File 1. Briefly, AGES-RS is a population-based cohort from Reykjavik, Iceland. Baseline data were collected from 2002 to 2006, with a second assessment approximately five years later.^12^ The CARDIA study is a US community-based, 4-centre longitudinal cohort of bi-racial individuals, who have been examined up to nine times since 1984 when they were aged 18–30 years old.^5^^,^^13^ DNAm data were collected at four-time points; in the 15th (Y15), 20th (Y20), 25th (Y25) and 30th (Y30) year follow-up exams, with n = 1568 samples available at baseline (Y15). The InCHIANTI study is a population-based cohort of residents aged 21–95 from villages in the Chianti region of Italy,^14^ with baseline phenotypic and DNAm data collected in 1998 for n = 678 individuals and follow-up assessments at 2007 and 2013, providing three time points.

### DNA methylation assay and quality control

Details of DNA methylation (DNAm) assays, quality control (QC) procedures, and the number of CpG sites and individuals remaining after QC for each cohort are provided in Supplementary File 1. In AGES-RS and CARDIA, DNA was extracted from whole blood and DNAm was measured using Illumina's Infinium MethylationEPIC BeadChip (∼850,000 CpGs), whereas in InCHIANTI, DNAm was assessed using the Illumina HumanMethylation450 BeadChip (∼450,000 CpGs) (Supplementary File 1).

### Estimation of the pace of ageing

We estimated the pace of BA using the DDPACE clock, designed to capture multi-organ ageing, implemented via an R command available on GitHub.^1^ Unlike previous epigenetic clocks, this clock was trained on longitudinal data acquired from a single-year birth cohort (born between 1972 and 1973). Reflecting longitudinal organ ageing, DDPACE provides a measure of the pace of BA relative to chronological age.^1^ For comparison, we also estimated BA using different principal component (PC)-based epigenetic clocks, PC-Horvath,^3^ PC-Hannumn,^15^ PC-GrimAge,^2^ and PC-PhenoAge,^4^ using R commands provided by Levine et al. lab.^16^

### Phenotypic data

We included seven CV health-related factors that modulate risk for disease, as proposed by the American Heart Association's Life's Simple 7.^17^ These factors include, smoking status, PA, diet, BMI, and biometric measures (systolic and diastolic blood pressure [SBP, DBP], total cholesterol, and fasting blood glucose levels). Details on the collection of these variables in each cohort are provided in Supplementary File 1. Due to the absence or limited availability of dietary data, the adapted-LS7 scores for the AGES-RS was estimated using our previously published algorithm based on six CV health-related factors,^18^ with a maximum achievable score of 12. For the CARDIA and InCHIANTI cohorts, the adapted-LS7 score was calculated using all seven factors, with a maximum possible score of 14. PA levels were assessed through a questionnaire, but the questions differed across the cohorts; specifics of PA questions by cohort are found in Supplementary File 2. We additionally included pack-years of smoking as a predictor, estimated using a DNAm-based metric as described by Lu et al.^19^

### Analytical sample

Of the total baseline AGES-RS cohort (n = 5764), DNA methylation was collected from n = 2602 participants. Of these, n = 2081 returned for the follow-up visit, had a second measure of DNAm. The 1998 InCHIANTI baseline assessment included n = 678. For the analyses based on two DDPACE scores, we created three subsets with samples from the following: 1998 and 2007 exams (n = 637), 2007 and 2013 exams (n = 344), and 1998 and 2013 exams (n = 364). For CARDIA, the ‘baseline’ Y15 DNAm subset included n = 1568 participants. For the analyses requiring two DDPACE measures, we created 4 subsets: Y15 and Y20 exams (n = 1395), Y20 and Y25 exams (n = 1697), Y25 and Y30 (n = 2464), and Y15 and Y30 (n = 1393). Our analyses were restricted to participants with complete data on both exposure and outcome variable; individuals with missing data for either were excluded.

### Statistical analyses

#### Descriptive

Descriptive statistics of all three cohorts were based on means (SD) for normally distributed continuous variables that met relaxed thresholds for normality (skewness between −2 and 2 and kurtosis between −7 and 7) and as medians and interquartile ranges (IQRs) for non-normally distributed variables. Categorical variables are described as counts and percentages.

#### Defining levels and changes in the DDPACE score

**Fig. 1:**
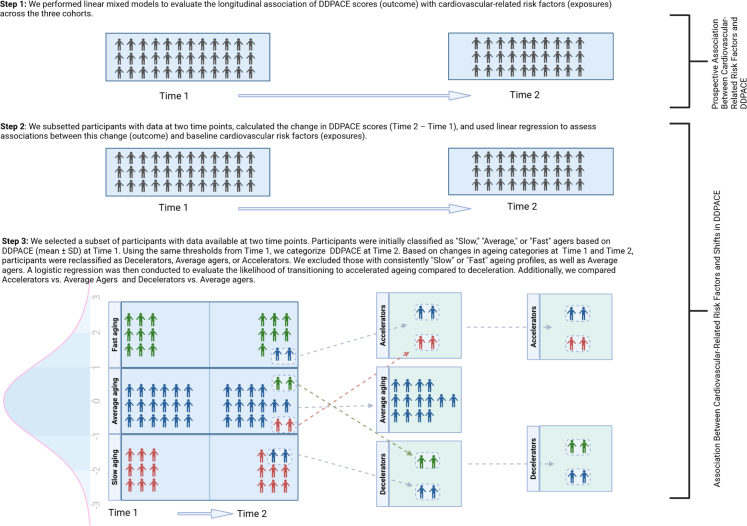
Flowchart summarizing the key analysis steps. In Step 1, linear mixed modeling was used to assess the longitudinal association between cardiovascular-related risk factors and the pace of ageing over time across the three cohorts. In Step 2, a change in DunedinPACE score (delta) was calculated between Time 1 and Time 2, and linear regression analyses was applied to examine prospective associations between baseline cardiovascular risk factors and changes in DunedinPACE scores. In Step 3, participants were categorized as ‘Slow,’ ‘Average,’ or ‘Fast’ agers at two time points using mean ± SD thresholds of the DunedinPACE score, with Time 1 thresholds applied consistently across both assessments. Based on their ageing pace at baseline and follow-up, participants were then reclassified as Decelerators, Average agers, or Accelerators. Logistic regression analyses were subsequently conducted to examine the associations between cardiovascular-related risk factors and the odds of shifting to an accelerated pace of ageing.

We conducted three main analyses (Fig. 1)^1^: examined the prospective longitudinal association between CV health-related risk factors (exposure) and the DDPACE score^2^; assessed the association between these risk factors and changes in DDPACE over time; and^3^ analysed categorical changes in DDPACE among participants who experienced a *shift* of ≥1 standard deviation (SD) from the mean.

For the *shift* analysis, we first classified participants at each time point (baseline and follow-up) into three groups based on the cohort-specific baseline mean and SD of DDPACE: "Slow" ageing (≥1 SD below the mean), "Average" ageing (within ±1 SD), and "Fast" ageing (≥1 SD above the mean) (Supplementary Fig. 1A–C). Using these classifications, we created nine baseline/follow-up combinations to track changes in ageing pace over time (e.g., Fast–Fast, Fast–Average, Average–Slow, etc.).

Due to none or small cell sizes in some of these categories, we collapsed them into three broader transition groups: Decelerators: individuals who shifted from a faster at baseline to a slower ageing pace at follow-up (e.g., fast–average or average–slow); Average Agers: individuals who remained in the average category at both time points; Accelerators: individuals who shifted from a slower to a faster pace (e.g., slow–average or average–fast).

### Statistical testing

#### Prospective longitudinal association of cardiovascular risk factors with DDPACE score

Details of the analytical approaches are provided below and illustrated in Fig. 1. We used linear mixed models to assess associations between CV health-related factors, including a composite risk score, and DDPACE scores at baseline and follow-up. Risk factors were modeled as time-varying to account for changes over time (Supplementary File 3, Equation 1). We developed a separate linear mixed model for each CV-related risk factor and calculated the beta coefficients (95% confidence intervals; 95% CI). To explore long-term associations, we also examined midlife CV risk factors (mean age 50) in relation to late-life DDPACE (mean age 76) in the AGES-RS cohort (n = 2602; Supplementary File 3, Equation 2). For comparison, we performed similar analyses in the AGES-RS cohort using BA scores from PC-based clocks (Horvath,^3^ Hannum,^15^ GrimAge,^19^ and PhenoAge^4^).

#### Change in DDPACE score

We applied linear regression (Supplementary File 3, Equation 3) to examine associations between Time 1 CV risk factors and change in DDPACE (Time 2 minus Time 1). We applied similar analyses in the AGES-RS cohort using BA change from PC-Horvath, PC-Hannum, PC-GrimAge, and PC-PhenoAge as the outcome.

#### Shift in DDPACE score

Logistic regression was used to assess associations between baseline CV risk factors and shifts in pace of ageing, estimating odds ratios (ORs) with 95% CIs. We focused on individuals who transitioned to accelerated or decelerated ageing, excluding those with consistent profiles across time points (Supplementary Fig. 1A–C). The primary comparison was between accelerators and decelerators, with secondary comparisons including accelerators vs. average agers and decelerators vs. average agers (Supplementary File 3, Equation 4).

We adjusted all models for the effects of sex, chronological age, educational level, white blood cell proportions, and assay batch. The change (delta) analysis was additionally adjusted for baseline DDPACE. Additionally, we included antihypertensive, lipid-lowering, and glucose-lowering medications when modeling BP, total cholesterol, and fasting plasma glucose, respectively. In addition, race and field centres were included as covariates for the CARDIA study. All analyses were conducted using R version 4.4.2. For ease of comparing beta coefficients, all continuous variables, including DDPACE, were converted to standard deviation (SD) units. Statistical significance was set at two-sided P-value <0.05. Analyses were the same for all three cohorts.

Based on the median timing of consecutive exams, we conducted two sets of analyses: one for ∼5-year follow-up intervals across all cohorts (AGES-RS: 2006–2011; CARDIA: Y15–Y20, Y20–Y25, and Y25–Y30, InCHIANTI: 2007–2013), and another for 9^+^ year intervals using CARDIA (Y15–Y30) and InCHIANTI (1998–2007,1998–2013) (Supplementary File 1). Fixed-effects meta-analyses summarized the multiple intervals within CARDIA and InCHIANTI, while random-effects models were used to combine results across all three cohorts.

### Ethics statement

The AGES-Reykjavik Study was approved by the National Bioethics Committee of Iceland (VSN: 00–063). All participants provided informed consent, and the study followed the committee's ethical guidelines. The CARDIA Study was approved by the Institutional Review Boards at all participating sites. Informed consent was obtained from all participants at each examination cycle. The present CARDIA study was approved under protocol IRB-990825030, issued by the CARDIA Single Institutional Review Board at the University of Alabama at Birmingham on March 2, 2023, covering the collection and use of all CARDIA data. The InCHIANTI Study was approved by the local ethics committee of the Italian National Institute of Research and Care on Ageing (protocol14/CE, 28 February 2000 and protocol 45/01, 16 January 2001). Participants provided written informed consent for study procedures and biological sample collection.

### Role of funders

Funders had no role in study design, data collection, data analyses, interpretation, or writing of the manuscript.

## Results

### Descriptive

Baseline characteristics of the three cohorts are shown in Table 1, and per study exam in Supplementary table S1. Baseline mean (SD) age in AGES-RS was 76.3 (5.2) years; range: 66.8–94.4, and 57.65% were women; in InCHIANTI, the mean (SD) age was 62.9 (15.7) (range: 21–95), and 55.6% were women, and in CARDIA, the mean (SD) baseline age at Y15 was 40.7 (3.6); range: 32.6–47.1) and 65.6% were women. CARDIA had a lower baseline DDPACE (SD) score (0.92 (0.13)) compared to InCHIANTI (1.06 (0.12)) and AGES-RS (1.10 (0.11)).

**Table 1:** Participant characteristics at DNAm baseline assessments across the three cohorts.

Characteristics	Study		
	AGES-RS (2006)	InCHIANTI (1998)	CARDIA (Y15)
Total number of participants, N	2602	678	1568
Sex (women, %)	1496 (57.5)	377 (55.6)	1030 (65.6)
Chronological age (years), mean (SD)	76.3 (5.2)	62.9 (15.7)	40.7 (3.6)
**Race, n (%)**^a^			
White, not Hispanic	–	–	864 (55.1)
Black, not Hispanic	–	–	702 (44.7)
Others	–	–	3 (0.2)
**Pace of biological age**			
DunedinPACE score, mean (SD)	1.10 (0.11)	1.06 (0.12)	0.92 (0.13)
**Modifiable behaviors**			
*Smoking status* (n (%))			
Never-smokers	1134 (43.6)	384 (56.6)	952 (60.7)
Former smokers	1167 (44.9)	159 (23.5)	309 (19.7)
Current smokers	296 (11.4)	135 (19.9)	305 (19.4)
Pack-years of smoking, mean (SD)^b^	15.44 (7.4)	8.52 (8.9)	7.45 (9.4)
*Moderate-to-vigorous PA*			
Never	1084 (41.7)	NA	NA
Poor	NA	60 (8.8)	289 (18.4)
Low	616 (23.7)	NA	NA
Moderate	447 (17.2)	275 (40.6)	535 (34.1)
High	420 (16.1)	339 (50)	740 (47.2)
PA total intensity score in Z-scores, median (IQR)^c^	−0.6 (0.6)	0.8 (0.9)	−0.2 (1.3)
History of healthy diet intake (n (%))			
Poor	NA	157 (23.3)	735 (52.7)
Medium	NA	305 (45.2)	631 (45.5)
High	NA	213 (31.6)	30 (2.1)
**Biometrics**			
BMI (kg/m^2^), mean (SD)	26.98 (4.3)	27.18 (3.9)	28.71 (7.1)
SBP (mmHg), median (IQR)	139 (24.3)	140 (28.8)	110 (17)
DBP (mmHg), median (IQR)	73 (12)	80 (100)	73 (14)
Fasting plasma glucose (mg/dL), median (IQR)	99.1 (12.6)	88 (15)	84 (11)
Total cholesterol (mg/dL), mean (SD)	217.48 (44.3)	216.13 (39.8)	183.68 (34.6)
**Medication history**			
Antihypertensive medication (Yes, n (%))	1212 (46.6)	211 (31.1)	114 (7.3)
Lipid-lowering medication (Yes, n (%))	615 (23.6)	31 (4.6)	35 (2.2)
Oral hypoglycemic medication (Yes, n (%))	151 (5.8)	34 (5)	37 (2.4)
**Composite CV risk factors**			
Adapted-LS7, median (IQR)^d^	7 (3)	8 (3)	9 (3)

PA: Physical activity; SBP: Systolic Blood Pressure; DBP: Diastolic Blood Pressure; BMI: Body Mass Index.

a The AGES-RS and InCHIANTI study populations are consisted of White, non-Hispanic individuals.

b Pack-years of smoking were estimated using a DNA methylation metric, Lu AT et al. *Aging (Albany NY)*. 2019; 11(2):303–327.

c Full description of PA variables in each cohort is provided in Supplemental File 2.

d Due to a lack of or limited diet data, the Adapted-LS7 scores for the AGES-RS cohort were estimated based on six cardiovascular-related factors, with a maximum achievable score of 12. In contrast, for the InCHIANTI and CARDIA cohorts, LS7 scores are based on a total of 14 scores.

### Prospective longitudinal association of pace of ageing with CV-risk factors

**Fig. 2:**
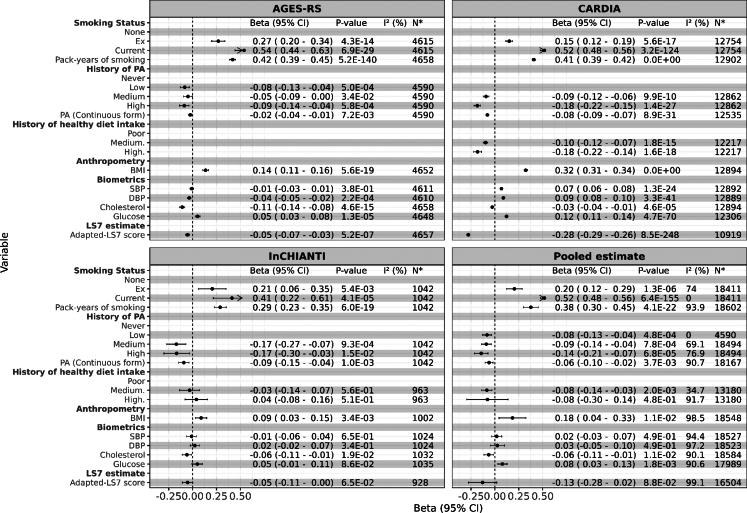
Longitudinal associations between cardiovascular-related factors and DunedinPACE scores over a 5-year follow-up across the three cohorts: AGES-RS, CARDIA, and InCHIANTI, with a meta-analysis of the three cohorts. All P-values were derived from two-sided linear mixed model regression analyses, and the P-values reported for the meta-analysis represent pooled P-value estimates across the three cohorts. All models were adjusted for sex, chronological age, white blood cell composition, educational level, and batch effects. Additionally, we adjusted the BP, cholesterol, and glucose models for the use of relevant medications. Models for the CARDIA cohort were further adjusted for race and data collection centre. PA: Physical activity; SBP: Systolic Blood Pressure; DBP: Diastolic Blood Pressure; BMI: Body Mass Index; LS7: Adapted Life’s Simple 7. Please refer to Supplementary File 2 for definitions of PA. N∗ is the number of observations in each model; in mixed models or meta-analyses, N∗ exceeds the number of unique samples.

Over a 5-year period, current and former smoking, more pack-years of smoking, BMI and blood glucose were associated with significantly higher DDPACE in all 3 cohorts and in the meta-analysis of the three cohorts (P < 0.05 (two-sided, linear mixed modeling)) (Fig. 2). History of PA, healthy diet intake, and higher levels of adapted-LS7 scores were associated with lower DDPACE. Cholesterol was inversely associated with DDPCE in all 3 cohorts (P < 0.05 (two-sided, linear mixed modeling)). The relationship between the BP and the DDPACE of ageing varied across the cohorts. Findings from the 9+ year follow-up period were consistent with those observed at the 5-year follow-up (Supplementary Figure 2).

Consistent with the findings for DDPACE, smoking status, PA (in both categorical and continuous forms), and cholesterol levels were associated with epigenetic age acceleration as measured by the PCPhenoAge, PCGrimAge, PCHannum, and PCHorvath clocks in the AGES-RS cohort, although effect sizes were smaller (Supplementary Table 3A). Findings from the long-term AGES-RS midlife analyses (mean age ∼50) were similar to those from the 5-year follow-up (Supplementary Figure 3).

### Change in DDPACE (time 2-time 1)

In the descriptive analyses, men exhibited greater changes in DDPACE compared to women in the CARDIA cohort; however, this sex difference was not observed in the other cohorts (Supplementary Tables 4A and 4B).

**Fig. 3:**
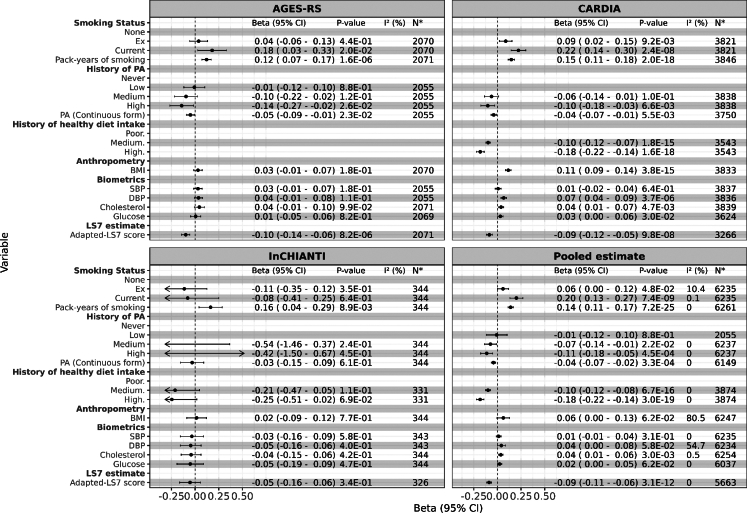
Association between 5-year changes in DunedinPACE (Time 2-Time 1) and cardiovascular health-related risk factors across three cohorts: AGES-RS, CARDIA, InCHIANTI, and a meta-analysis combining all three cohorts. All P-values were derived from two-sided linear regression analyses, and the P-values reported for the meta-analysis represent pooled P-value estimates across the three cohorts. All models were adjusted for sex, baseline chronological age, white blood cell composition, baseline educational level, baseline DunedinPACE, and batch effects. Additionally, we adjusted the BP, cholesterol, and glucose models for the use of relevant medications at baseline. Models for the CARDIA cohort were further adjusted for race and data collection centre. PA: Physical activity; SBP: Systolic Blood Pressure; DBP: Diastolic Blood Pressure; BMI: Body Mass Index; Adapted-LS7: Adapted Life’s Simple 7. Please refer to Supplementary File 2 for definitions of PA. N∗ represents number of observations in each model; in meta-analyses, N∗ accounts double counting.

Patterns of association between baseline CV-related factors and 5-year change in DDPACE were largely consistent with the 5-year longitudinal analysis (Fig. 3), except in InCHIANTI cohort. The strongest factor that was highly significantly associated with decreased DDPACE was the adapted-LS7 (P = 2.41 × 10^−11^ (two-sided, linear regression)) in the meta-analyses of the three cohorts (Fig. 3). A history of high healthy diet intake was also associated to lower DDPACE in the pooled estimate of the CARDIA and InCHIANTI cohorts (P = 3.00 × 10^−19^ (two-sided, linear regression)). The 9+ years results differed between the CARDIA and InCHIANTI cohorts (Supplementary Figure 4). In CARDIA, associations of CV-related risk factors with DDPACE change were directionally similar to the 5-year data (Fig. 3). In the meta-analysis of the CARDIA and InCHIANTI cohorts, only current smoking and pack-years of smoking were associated with greater increases in DDPACE change over the 9+ years of follow-up, whereas only PA was associated with a decrease in DDPACE change.

In the AGES-RS cohort, current smoking and pack-years of smoking were associated with increases in PC-Horvath and PC-PhenoAge change, but PC-GrimAge change showed an inverse relationship with pack-year of smoking (Supplementary Table 3B). Baseline adapted-LS7 was associated with a marginal decrease (P > 0.05 (two-sided, linear regression)) in BA change across all clocks.

### Odds of shifting to an accelerated or decelerated pace of ageing

Among the 2081 AGES-RS participants, 25.4% (n = 528) showed DDPACE shifts: 6.0% (n = 125) decelerated and 19.4% (n = 403) accelerated (Supplementary Figure 1A). In CARDIA, more decelerated (9.2%) and fewer accelerated (15.5%) than in AGES-RS (Supplementary Figure 1B). InCHIANTI showed a similar pattern to AGES-RS (Supplementary Figure 1C). Distribution of baseline CV-related risk factors (e.g., former and current smokers) were higher in accelerators than decelerators (Supplementary Table 2).

**Fig. 4:**
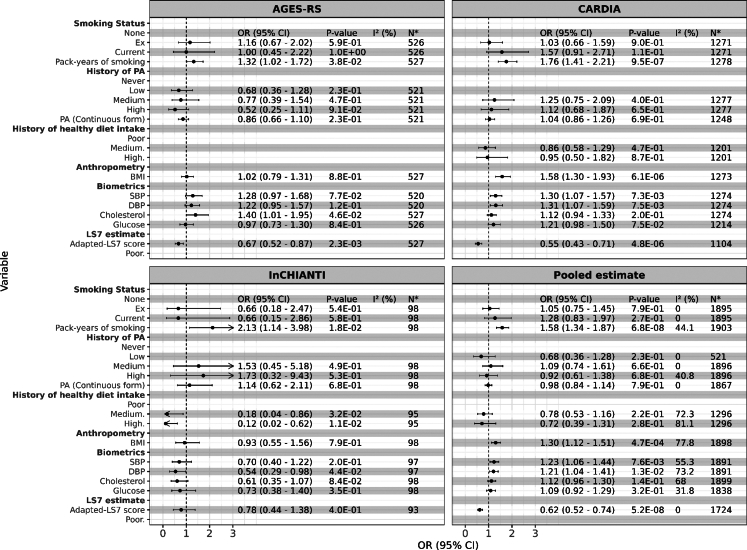
The odds ratios (ORs, 95% CIs) for shifting to an accelerated pace of aging compared to decelerated aging in the AGE-RS, CARDIA, InCHIANTI and meta-analysis of the three cohorts in a 5-year interval. All P-values were derived from two-sided logistic regression analyses, and the P-values reported for the meta-analysis represent pooled P-value estimates across the three cohorts. All models were adjusted for sex, baseline chronological age, white blood cell composition, baseline educational level, baseline DunedinPACE, and batch effects. Additionally, we adjusted the BP, cholesterol, and glucose models for the use of relevant medications at baseline. Models for the CARDIA cohort were further adjusted for race and data collection centre. PA: Physical activity; SBP: Systolic Blood Pressure; DBP: Diastolic Blood Pressure; BMI: Body Mass Index; Adapted-LS7: Adapted Life’s Simple 7. Please refer to Supplementary File 2 for definitions of PA. N∗ is the number of observations in each model; in meta-analyses, N∗ accounts double counting.

Fig. 4 presents the ORs (95% CIs) for shifting to an accelerated pace of ageing compared to a decelerated pace. The results were consistent between the AGES-RS and CARDIA cohorts over a 5-year period. In the meta-analysis of the 5-year interval data, more pack-years of smoking, higher BMI, SBP, and DBP were associated with a greater odds of being in the accelerator compared to decelerator group (OR >1, P < 0.05 (two-sided, logistic regression)), while the adapted-LS7 was associated with a higher likelihood of being in the decelerator group (OR < 1, P < 0.05 (two-sided, logistic regression)). Over the 9^+^-year interval, results differed between CARDIA and InCHIANTI, but no significant association was observed in the pooled analysis (Supplementary Figure 5). In the analysis comparing accelerated vs. average ageing in the 5-year follow-up, smokers and individuals with higher BP and blood glucose levels were more likely to experience acceleration in their pace of ageing (Supplementary Figure 6). In the 9^+^-year follow-up, only smokers showed increased odds of accelerated ageing (Supplementary Figure 7). Comparisons of decelerators vs. average agers are shown in Supplementary Figures 8 and 9 for the 5-year and 9^+^-year follow-up periods, respectively.

## Discussion

In this analysis, we provided findings on the short- and longer-term magnitude of change in the DDPACE of ageing, and how CV health-related risk factors are associated with these changes. We found that individual CV health-related risk factors, as well as a composite score, were associated with changes in DDPACE over 5-year and 9^+^-year follow-up periods, accounting for the changes in the exposure levels.

Although our prospective analyses findings are consistent with the literature on healthy hearts,^5^^,^^20^ individual risk factors were variably associated in direction and significance of association across cohorts and exams when focusing on individuals who showed DDPACE shifts. High pack-years of smoking, higher BMI, SBP, and DBP were associated with increased odds of shifting to an accelerated pace of ageing, whereas healthy diet and higher adapted-LS7 scores were clearly associated with a shift in DDPACE toward a slower ageing pace. Our life course analysis (∼26-year follow-up from midlife) also shows that the magnitude of the association of DDPACE with smoking, BMI, glucose, and adapted-LS7 was similar to that observed within a shorter follow-up period of 5 years in the AGES-RS cohort, but the strength of the evidence for different levels of PA was weaker in the longer follow-up period (P > 0.05 (two-sided, linear mixed modeling)).

Our findings are consistent with previous studies showing that CV factors, both individually and collectively, are associated with BA, as measured by GrimAge, Horvath, and Hannum clocks, in both cross-sectional analyses and longitudinal studies over follow-up periods exceeding 10 years.^5^^,^^21^, ^22^, ^23^ This trend has also been seen in younger participants, adolescents aged 12 to 17, in the longitudinal FinnTwin12 study, who were categorized into five classes based on their lifestyle behaviour patterns (e.g., smoking status, alcohol consumption, and PA history). Those in the unhealthiest lifestyle classes exhibited faster DDPACE, PhenoAge, and GrimAge ageing compared to those in healthier lifestyle classes, and this difference persisted into young adulthood (ages 21–25).^24^ Other smaller studies, one involving 6 women in a case series^8^ and the other involving 38 men in a clinical trial,^10^ demonstrated a reduction in BA using the Horvath clock following diet and lifestyle interventions within an 8-week follow-up.

The adapted-LS7 score was consistently linked to slower ageing in longitudinal analyses across all three cohorts (Figs. 2 and 3) and in the life course analyses of the AGES-RS cohort (Supplementary Figure 3). Furthermore, individuals with higher adapted-LS7 scores were less likely to shift into accelerated pace of ageing (Fig. 4). The strength and consistency of the association between the composite adapted-LS7 and a slower pace of ageing may suggest that a more comprehensive approach to CV health may offer benefit in slowing BA and potentially reducing the risk of frailty and mortality.^25^ We found specific behavioural factors and estimates of effect durability that can be adhered to slow ageing, notably no cigarette smoking, taking healthy diet, and engaging in some level of PA.

Our longitudinal and change association analyses confirmed the well-established link between smoking and pace of ageing.^26^^,^^27^ Specifically, pack-years of smoking, and both former and current smokers exhibited a faster pace of biological ageing across the three cohorts in a dose–response manner (Figs. 2 and 3). However, when focusing on individuals who exhibited shifts in DDPACE (excluding those with consistently on average, fast, or slow ageing pace), only pack-years of smoking was associated with a higher likelihood of shifting to an accelerated ageing pace, compared to a decelerated pace (Fig. 4 and Supplementary Figure 5). These findings may suggest that the associations observed for former and current smoking may reflect the cumulative and long-term effects of smoking. This is further supported by our long-term follow-up results from the AGES-RS cohort, where both former and current smoking at midlife were significantly associated with late-life DDPACE (Supplementary Figure 3). Studies suggest that smokers may be able to reverse DNA methylation changes in some CpG sites,^28^ while other findings argue that the reversal effects are specific to certain sites, with smoking still exerting a lasting impact on DNA methylation.^11^ However, these studies primarily focused on DNA methylation alterations without adequately addressing how these changes translate into changes in the pace of ageing.

The lasting effects of PA are less clear as the results from 5-year follow-up shift study (Fig. 4) were not consistent with the 5-year prospective and change analyses (Figs. 2 and 3), and midlife (Supplementary Figure 3). However, the questions ascertaining PA differed amongst the cohorts, so this variable had relatively more error than the other variables. Previous studies have shown that individuals who engage in PA have altered DNAm compared to those who were inactive,^29^^,^^30^ but the question of whether the changes in methylation are sustained has not been investigated.

Higher BMI and glucose levels were longitudinally associated with a faster pace of ageing, whereas higher total cholesterol levels were linked to a slower pace of ageing across three cohorts (Fig. 2). However, in the meta-analysis of the 5-year shift analyses, elevated BMI but not glucose was associated with a higher odds of shifting to accelerated pace (Fig. 4). The association of BP with DDPACE was inconsistent across cohorts and within the different analysis approaches.

There are several proposed mechanisms through which smoking may accelerate biological ageing. Evidence from multiple studies shows that smoking induces DNA methylation changes at specific CpG sites, particularly within genes involved in antioxidant defence and inflammation, such as AHRR, GPR15, and F2RL3.^31^^,^^32^ These epigenetic alterations are thought to contribute to cellular senescence, organ dysfunction, immunosenescence, and the development of age-related diseases.^33^^,^^34^ Furthermore, studies indicate that DNA methylation-based ageing measures (e.g., GrimAge, PhenoAge, and DunedinPACE) partly mediate the associations between smoking and cardiometabolic traits, cancer, and mortality, thereby supporting a mechanistic relationship from smoking to altered DNA methylation and ultimately to accelerated ageing phenotypes.^35^^,^^36^

Conversely, lifestyle interventions such as healthy diet and regular PA have been associated with slower biological ageing.^8^^,^^37^ Engaging in regular PA may attenuate pro-inflammatory methylation signatures, reduce oxidative stress, and enhance DNA repair, thereby counteracting key drivers of cellular ageing.^38^ For example, in a five-year follow-up of a population-based cohort, 60 min per day of light-intensity PA was associated with higher methylation of the *ASC* gene, a potential biomarker of systemic inflammation.^39^ Similarly, diet may influence DNA methylation through several mechanisms. These include directly supplying substrates and cofactors (e.g., folic acid, vitamin B12, betaine, and choline) required for methylation reactions, or indirectly modifying the activity of enzymes involved in one-carbon metabolism, thereby regulating the expression of genes associated with ageing.^40^, ^41^, ^42^ On the other hand, evidence such as the partial reduction of biological ageing observed 12 months after bariatric surgery,^43^ the association of BMI with DNA methylation at genes such as *THADA*^44^ (a glucose-regulating gene), and the causal link between a BMI-based methylation risk score and elevated levels of the inflammatory biomarker IL-6, may highlight how BMI can drive metabolic dysregulation and accelerated ageing.^44^

Our findings have significant research and clinical implications for understanding the impact of various CV factors on the pace of ageing in adults. Results of the ageing shift analyses, strengthens the established evidence regarding the relationship between lifestyle factors and epigenetic ageing. On the other hand, the midlife analyses results show the importance of quitting smoking and regular PA in potentially mitigating adverse ageing outcomes. The consistent association between the adapted-LS7 scores and the pace of ageing highlights the utility of this scoring system in predicting and assessing ageing trajectories. It suggests that interventions aimed at improving lifestyle and behavioural factors before midlife, as measured by the adapted-LS7 score, may have beneficial effects not only on CV health but also on overall ageing-related health outcomes.

A key strength of this study is the inclusion of both mid-life and late-life data in AGES-RS, allowing examination of both short- and long-term associations between CV risk factors and pace of ageing in the same population. Another strength is the use of longitudinal DNAm data to assess the dynamic nature of ageing, in contrast to prior cross-sectional studies.

However, there are also issues that need to be taken into account when interpreting the results. The AGES-RS and InCHIANTI cohorts included only White participants. While CARDIA included both Black and White participants and showed similar findings to AGES-RS, further research in more diverse populations is needed. Results in InCHIANTI showed some inconsistencies, likely due to its smaller sample size, particularly in accelerator and decelerator groups. InCHIANTI also spanned a wider age range, including both younger individuals, whose methylation profiles may still be changing, and older adults with more stable profiles.^6^ We used different methylation platforms across cohorts (Illumina 450k in InCHIANTI and MethylEPIC in AGES-RS and CARDIA), which could have influenced the results. To address this, we combined the three cohorts and adjusted the association analyses for both BeadChip and cohort. The associations between CV health-related risk factors and DDPACE remained essentially unchanged, indicating that platform differences did not affect our findings (Supplementary Table 5). Similarly, PA history was assessed using different questionnaires across the three cohorts: a validated, interviewer-administered instrument in CARDIA,^45^ a questionnaire based on U.S. Department of Health and Human Services recommendations^46^ in InCHIANTI, and a self-reported measure in AGES-RS. Although these tools are not directly comparable and may have introduced heterogeneity in PA measurement, the validated instruments in CARDIA and InCHIANTI, along with the consistent 12-month recall period in AGES-RS and CARDIA, may help harmonize PA-related data and strengthen the comparability of findings across cohorts.

In longitudinal analyses, total cholesterol was inversely associated with DDPACE across all cohorts, consistent with prior large studies.^2^^,^^47^ This may reflect the impact of statin use, which has been linked to DNA methylation changes^48^ and lower CV risk.^49^ Although specific statin data were unavailable, models were adjusted for any lipid-lowering medications, partially addressing this limitation.

Despite the longitudinal design, selective dropout may have introduced bias. In AGES-RS, 20% (n = 521/2602) dropped out; they were older at baseline (79.6 ± 5.2 vs. 75.5 ± 4.8 years, P < 0.05 (two-sided, independent T-test)) and had a higher DDPACE (1.11 ± 0.12 vs. 1.09 ± 0.11, P < 0.05 (two-sided, independent T-test)). In InCHIANTI, the largest dropout occurred between 2007 and 2013 (47.7%, n = 314/658); these participants were older (76.8 ± 14.0 vs. 67.7 ± 15.6 years, P < 0.05 (two-sided, independent T-test)) and had a higher DDPACE (1.13 ± 0.13 vs. 1.08 ± 0.13, P < 0.05 (two-sided, independent T-test)). In contrast, CARDIA expanded its sample with DNAm data collected at multiple exams: n = 1568 (Year 15), n = 1807 (Year 20), n = 2715 (Year 25), and n = 2597 (Year 30). Finally, we estimated BA from whole blood, which may limit accuracy, as methylation patterns vary across tissues. Evidence from prior animal and clinical trial studies have suggested BA can change over time.^9^ The concept of an epigenetic clock is based on hypothesis that specific sites in the genome undergo changes in DNA methylation with age that are progressive and common across individuals and sometimes even tissues. However, DNAm may vary by tissues and by organs even within individuals,^50^ potentially limiting the accuracy of ageing process estimates.

We demonstrated that CV-related factors (smoking, PA, BMI, fasting glucose, total cholesterol, and adapted-LS7 scores) were longitudinally associated with pace of ageing in midlife and older adults across the three cohorts. Among individuals who experienced substantial changes in their pace of ageing (shifts), the same factors were also linked to accelerated or decelerated ageing; however, these associations were less consistent across the three cohorts. Interventions to quit smoking, maintain regular exercise, as well as management of preventable and treatable CV risk factors starting at least by middle-age, may contribute to delays in ageing and reducing the burden of age-related diseases. Furthermore, our findings highlight the dynamic nature of BA over time, underscoring the limitations of cross-sectional data in epigenetic ageing studies.

## Contributors

**NGA:** Contributed to study conception and design, literature review, primary data analysis, and interpretation; integrated input from all co-authors; generated figures and led manuscript drafting and revisions. **LJL:** Contributed to study design, data analysis and interpretation, data acquisition; assisted in figure preparation and manuscript writing; made critical revisions and approved the final version for submission. **YH**: Contributed to data analysis and interpretation, provided critical review of the manuscript for important intellectual content, and approved the final version for submission. **ZL:** Contributed to data analysis, provided critical review of the manuscript for important intellectual content, and approved the final version for submission. **OM:** Assisted in data analysis and interpretation, provided critical review of the manuscript for important intellectual content, and approved the final version for submission. **JMR:** Contributed to data analysis and literature review; provided critical review of the manuscript for important intellectual content, and approved the final version for submission. **YZ:** Contributed to data analysis and interpretation, data acquisition; provided critical review of the manuscript for important intellectual content, and approved the final version for submission. **DLJ:** Contributed to data acquisition; provided critical review of the manuscript for important intellectual content, and approved the final version for submission. **LH:** Contributed to data acquisition; provided critical review of the manuscript for important intellectual content, and approved the final version for submission. **PK:** Contributed to data analysis and interpretation, data acquisition; provided critical review of the manuscript for important intellectual content, and approved the final version for submission. **TT:** Contributed to data analysis and interpretation, data acquisition; provided critical review of the manuscript for important intellectual content, and approved the final version for submission. **LF:** Contributed to data acquisition; provided critical review of the manuscript for important intellectual content, and approved the final version for submission. **ValG:** Contributed to data acquisition; provided critical review of the manuscript for important intellectual content, and approved the final version for submission. **VilG:** Contributed to data acquisition; provided critical review of the manuscript for important intellectual content, and approved the final version for submission. **TA:** Contributed to data acquisition; provided critical review of the manuscript for important intellectual content, and approved the final version for submission.

**NGA, ZL,** and **LJL** had full access to the AGES-RS data and access to the CARDIA and InCHIANTI data used in this manuscript; **LH** and **YZ** had full access to the CARDIA data; and **TT**, **LF**, and **PK** had full access to the InCHIANTI data.

## Data sharing statement

The data used in this study are available to qualified researchers upon reasonable request. Interested investigators should submit data access applications directly to the corresponding study cohorts, in accordance with their data sharing policies and procedures. For the AGES-RS and InCHIANTI study cohorts, data requests can be sent to djass.mbangdadji@nih.gov and tanakato@mail.nih.gov, respectively. For the CARDIA study, interested researchers can request data online at https://www.cardia.dopm.uab.edu/. R commands used in this study are available on GitHub: https://github.com/niguurayugenetics/Bioaging-shift-analysis-r-command-.

## Disclaimer

This research was supported [in part] by the Intramural Research Program of the National Institutes of Health (NIH). The contributions of the NIH author(s) are considered Works of the United States Government. The findings and conclusions presented in this paper are those of the author(s) and do not necessarily reflect the views of the NIH or the U.S. Department of Health and Human Services.

## Declaration of Generative AI and AI-Assisted Technologies in the Writing Process

During the preparation of this work the first author used ChatGPT and Claude AI in order to assist with language editing and to refine R commands for data analysis and figure generation. The author(s) have reviewed and confirmed the validity of the text and take(s) full responsibility for the content of the publication.

## Declaration of interests

The authors declare no competing financial interests or personal relationships that could have appeared to influence the work reported in this paper.
