# Supplementary material for: Mid- and late-life cardiovascular health indicators and changes in biological ageing Markers; A multi-cohort study
Source: eBioMedicine. 2025 Nov 11;122:106016. doi: 10.1016/j.ebiom.2025.106016 (PMC12657379; doi:10.1016/j.ebiom.2025.106016)
Supplement: Supplementary Figure 1 [file mmc1.docx]

**Supplementary
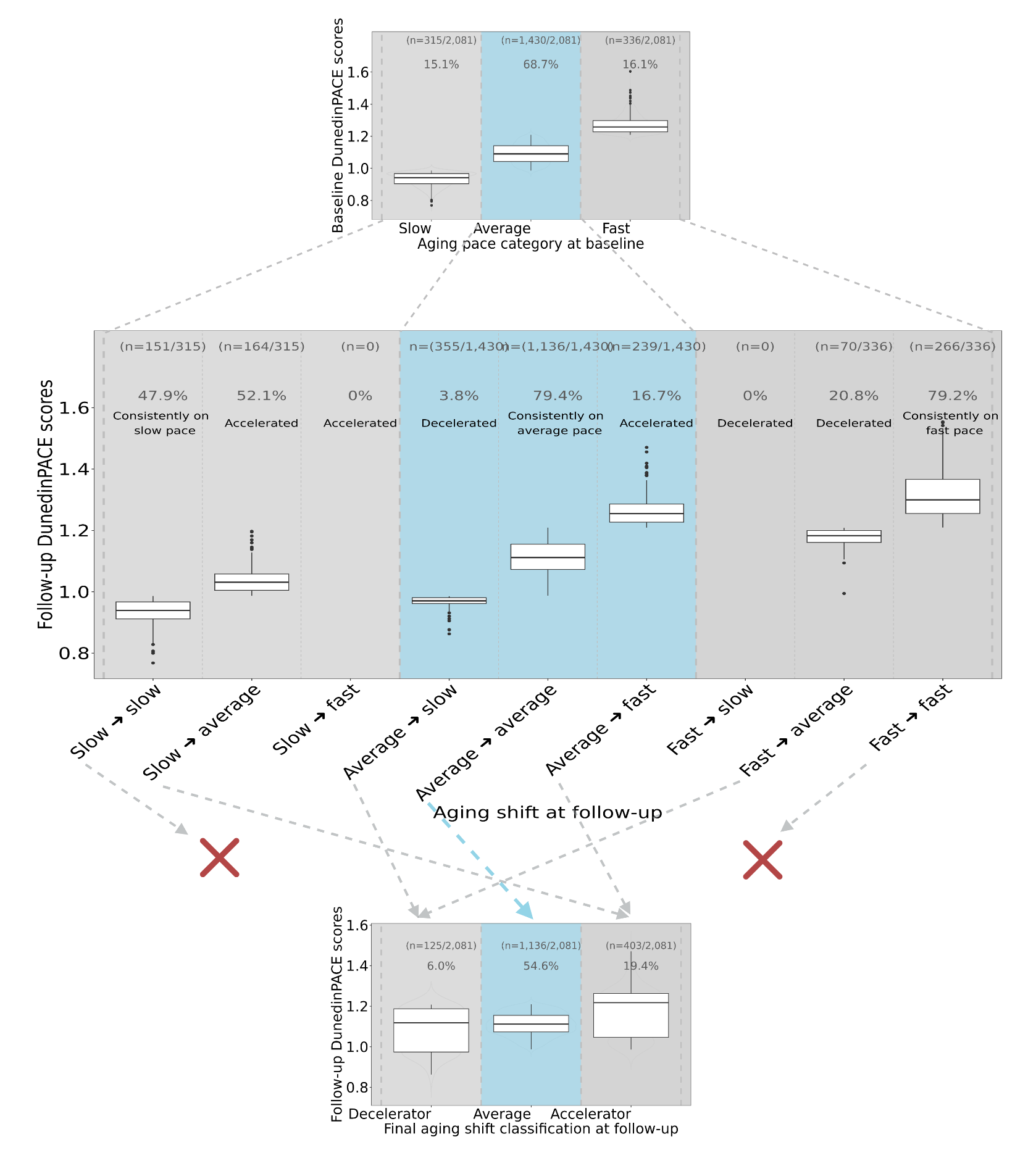
Figure 1. Distribution of participants across pace of ageing categories at baseline, shifts at follow-up, and final shift classifications in the AGES-RS cohort (a), CARDIA (b), and InCHIANTI (c) cohorts.**

a


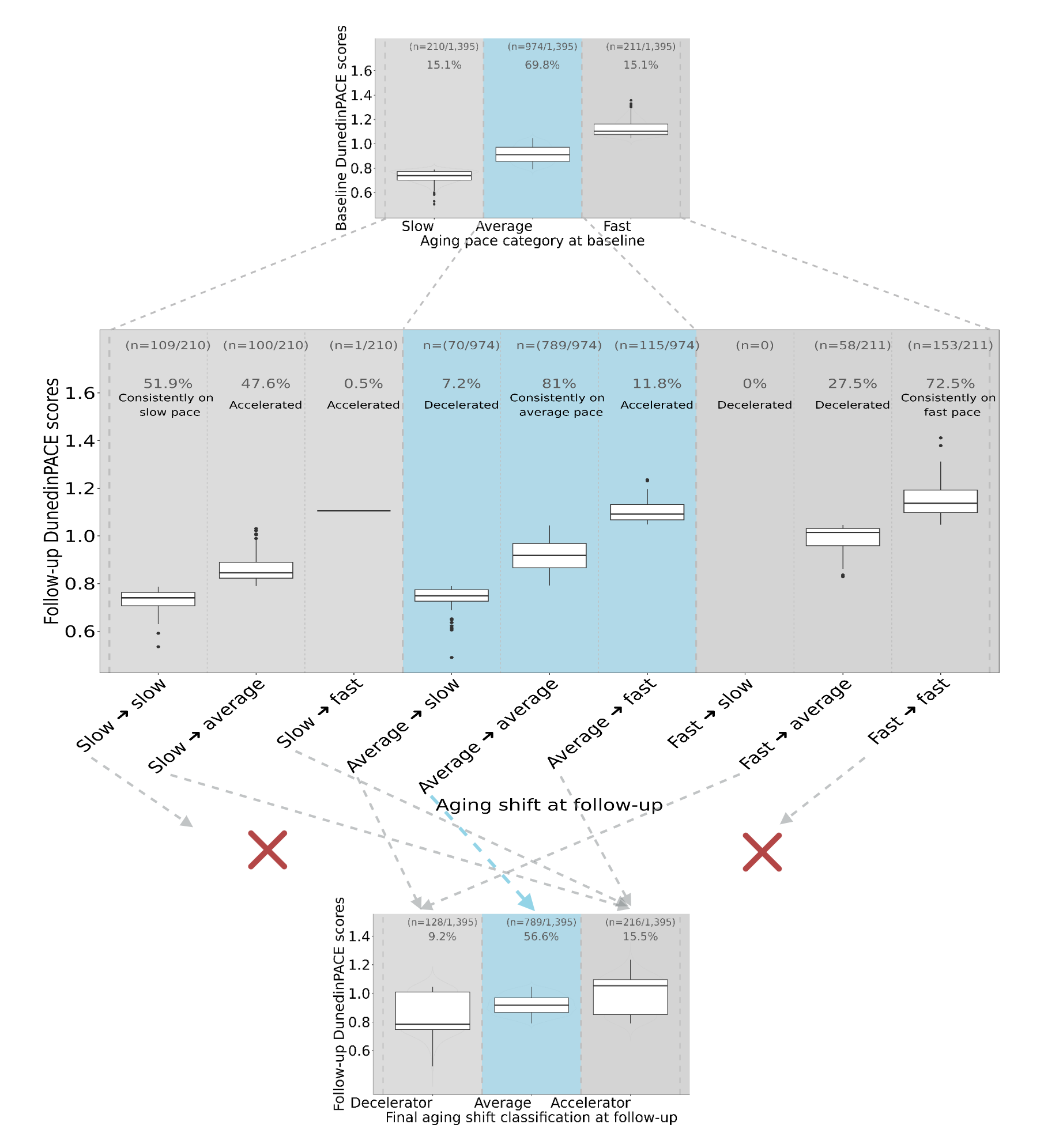


b


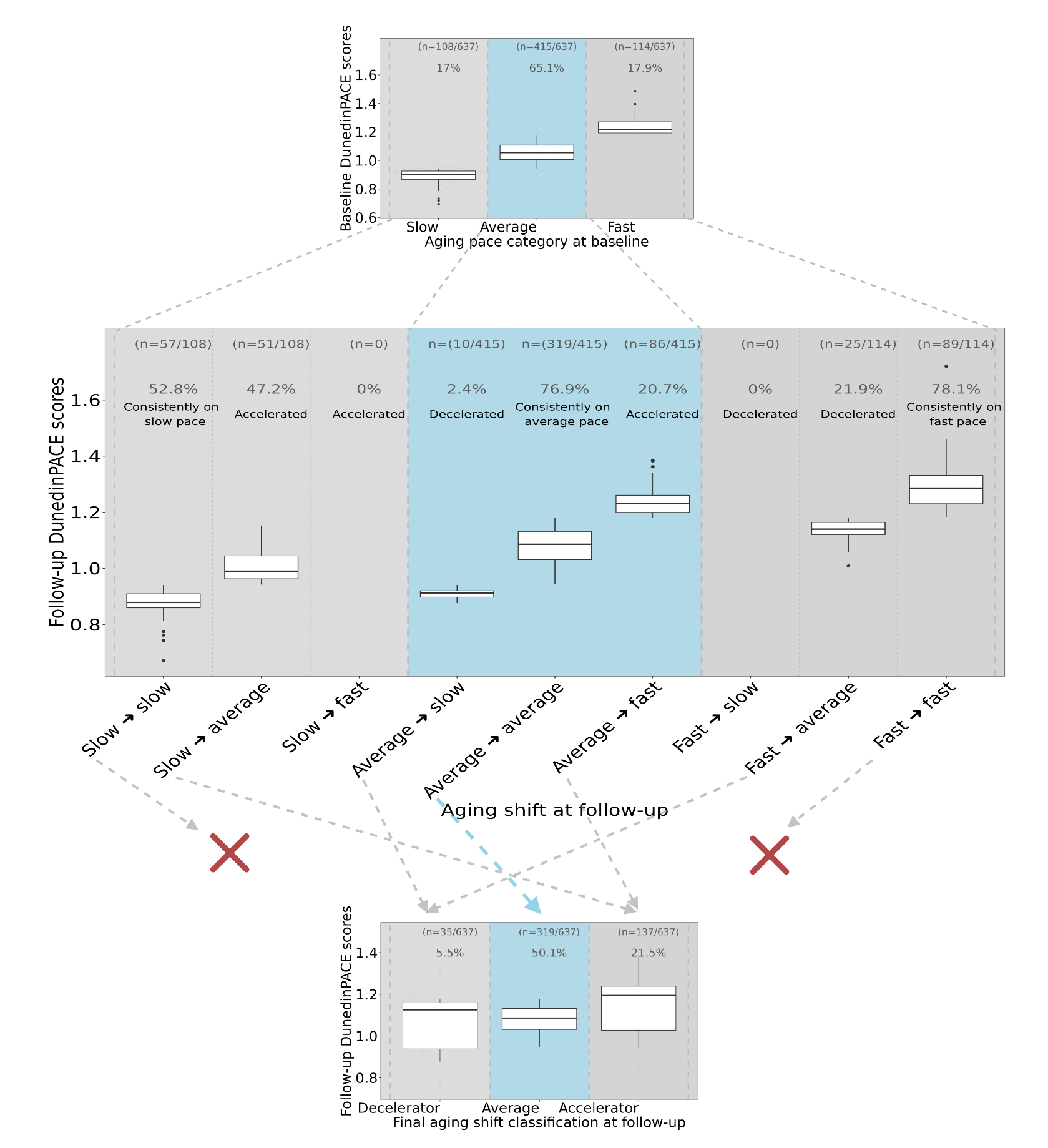


c

*Supplementary Figure 1 displays classification of participants into three ageing pace categories based on the DunedinPACE score at baseline: Slow (≤ mean − 1 SD), Average (within ±1 SD of the mean), and Fast (≥ mean + 1 SD). The same thresholds from baseline were applied to the follow-up assessment. Participants with consistently “slow” or consistently “fast” ageing profiles were excluded. The remaining participants were classified as “decelerators,” “average agers,” or “accelerators” based on changes in their ageing pace between baseline and follow-up. Results are presented for the AGES-RS (2006–2011), CARDIA (Year 15–20), and InCHIANTI (1998–2007) cohorts.*
