# Supplementary material for: Mid- and late-life cardiovascular health indicators and changes in biological ageing Markers; A multi-cohort study
Source: eBioMedicine. 2025 Nov 11;122:106016. doi: 10.1016/j.ebiom.2025.106016 (PMC12657379; doi:10.1016/j.ebiom.2025.106016)
Supplement: Supplementary Figure 2 [file mmc2.docx]

**Supplementary Figure 2. Longitudinal associations between cardiovascular health-related risk factors and pace of ageing (DunedinPACE) over a 9^+^-year follow-up in the CARDIA and InCHIANTI cohorts, as well as in the meta-analysis combining the two cohorts.**

**
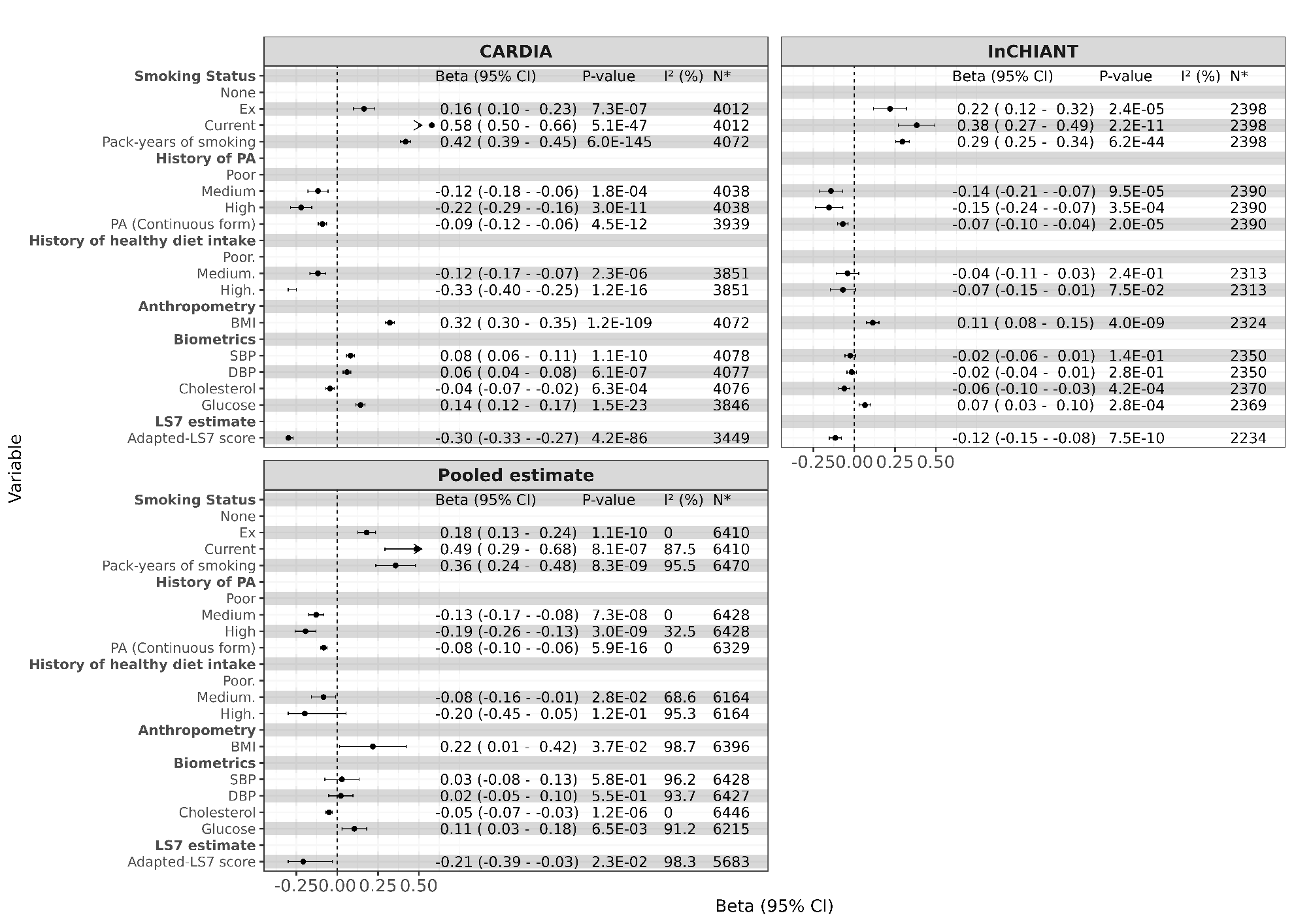
**

All ***p-values*** were derived from two-sided linear mixed model regression analyses, and the p-values reported for the meta-analysis represent pooled p-value estimates across the two cohorts. Supplementary Figure 2 shows longitudinal association between cardiovascular-related factors and ageing pace, estimated using DunedinPACE scores, across two cohorts: CARDIA and InCHIANTI, with pooled estimates of the two cohorts.

All models were adjusted for sex, chronological age, white blood cell composition, educational level, and batch effects. Additionally, we adjusted the BP, cholesterol, and glucose models for the use of relevant medications. Models for the CARDIA cohort were further adjusted for race and data collection center.

PA: Physical activity; SBP: Systolic Blood Pressure; DBP: Diastolic Blood Pressure; BMI: Body Mass Index; LS7: Adapted Life’s Simple 7.

Please refer to Supplementary File 2 for definitions of PA.

N* is the number of observations in each model; in mixed models or meta-analyses, N* exceeds the number of unique samples.
