# Supplementary material for: Mid- and late-life cardiovascular health indicators and changes in biological ageing Markers; A multi-cohort study
Source: eBioMedicine. 2025 Nov 11;122:106016. doi: 10.1016/j.ebiom.2025.106016 (PMC12657379; doi:10.1016/j.ebiom.2025.106016)
Supplement: Supplementary Figure 3 [file mmc3.docx]

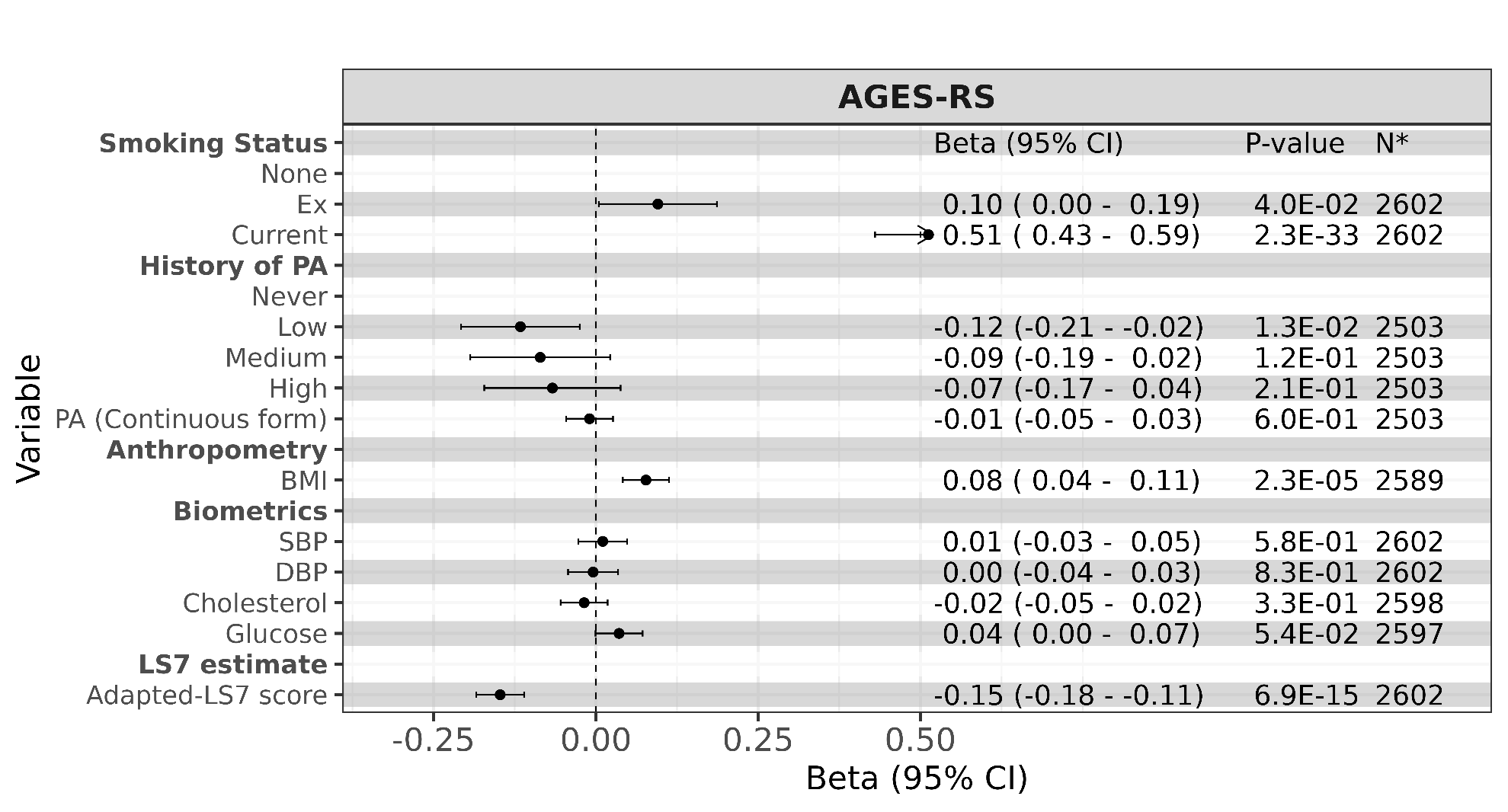
**Supplementary Figure 3. Associations of cardiovascular-related factors measured in midlife (mean age ~50 years) with DunedinPACE scores assessed at baseline (mean age ~76 years) in the AGES-RS cohort.**

All ***p-values*** were derived from two-sided linear regression analyses. Supplementary Figure 3 *presents the beta estimates from a linear regression model analysing the pace of ageing at follow-up in the AGES-RS cohort (average age ~76 years) using cardiovascular-related factors measured during midlife (average age ~50 years) as predictor variables. The models were adjusted for sex, midlife chronological age, white blood cell composition, midlife educational level, and batch. Additionally, we adjusted the BP, cholesterol, and glucose models for the use of relevant medications. PA: Physical activity; SBP: Systolic Blood Pressure; DBP: Diastolic Blood Pressure; BMI: Body Mass Index. History of moderate-to-vigorous physical activity in the last 12 months was categorized as follows: Never: (0 hrs/week); Low: (> 0 and <1.5 hrs/week); Moderate (≥ 1.5 and < 5.5 hrs/week); and High (≥ 5.5 hrs/week)**.* N* is the number of observations in each model.
