# Supplementary material for: Mid- and late-life cardiovascular health indicators and changes in biological ageing Markers; A multi-cohort study
Source: eBioMedicine. 2025 Nov 11;122:106016. doi: 10.1016/j.ebiom.2025.106016 (PMC12657379; doi:10.1016/j.ebiom.2025.106016)
Supplement: Supplementary Figure 4 [file mmc4.docx]

**Supplementary Figure 4. Association between cardiovascular health-related risk factors and 9^+^ years changes in DunedinPACE (Time 2 – Time 1) across the two cohorts; CARDIA, InCHIANTI, and meta-analysis of these two cohorts.**


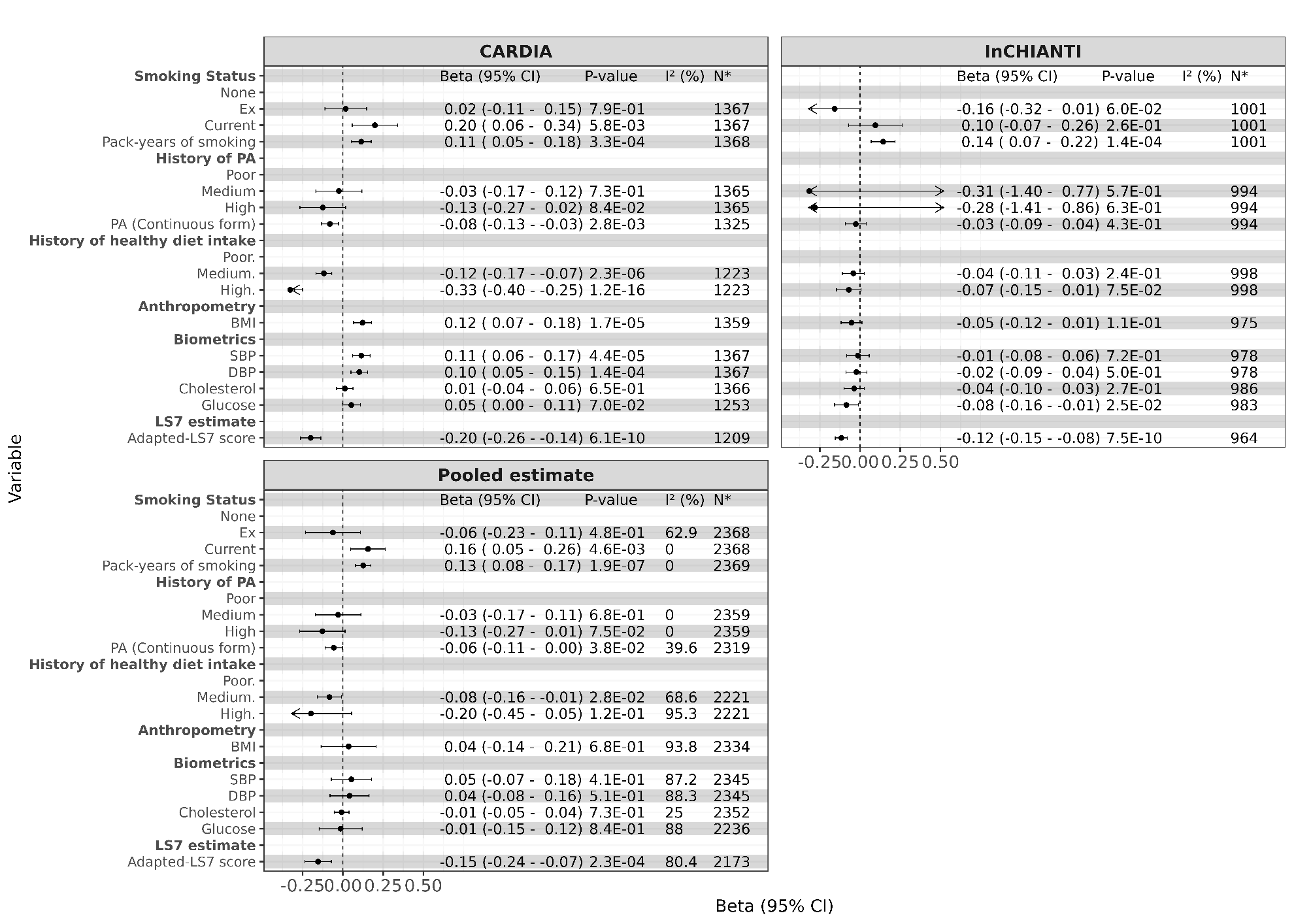


*All* ***p-values*** *were derived from two-sided linear regression analyses, and the p-values reported for the meta-analysis represent pooled p-value estimates across the two cohorts. Supplementary Figure 4 provides beta estimates derived from linear regression analysis. The outcome of interest was the difference in pace of ageing between two distinct time points, while the exposures were baseline cardiovascular-related factors. Individuals who had data at two distinct time points were included. The models were adjusted for sex, baseline chronological age, baseline DunedinPACE, white blood cell composition, baseline educational level, and batch. Furthermore, BP models were additionally adjusted for antihypertensive medications, cholesterol models were additionally adjusted for lipid-lowering medications, fasting glucose models were additionally adjusted for glucose lowering medications in all cohorts. All models in CRADIA cohort were additionally adjusted for race and data collection center.*

*PA: Physical activity; SBP: Systolic Blood Pressure; DBP: Diastolic Blood Pressure; BMI: Body Mass Index; LS7: Life’s Simple 7.*

*Please refer to Supplementary File 2 for definitions of PA.*

*N* is the number of observations in each model; in meta-analyses, N* exceeds the number of unique samples.*
