# Supplementary material for: Mid- and late-life cardiovascular health indicators and changes in biological ageing Markers; A multi-cohort study
Source: eBioMedicine. 2025 Nov 11;122:106016. doi: 10.1016/j.ebiom.2025.106016 (PMC12657379; doi:10.1016/j.ebiom.2025.106016)
Supplement: Supplementary Figure 6 [file mmc6.docx]

**
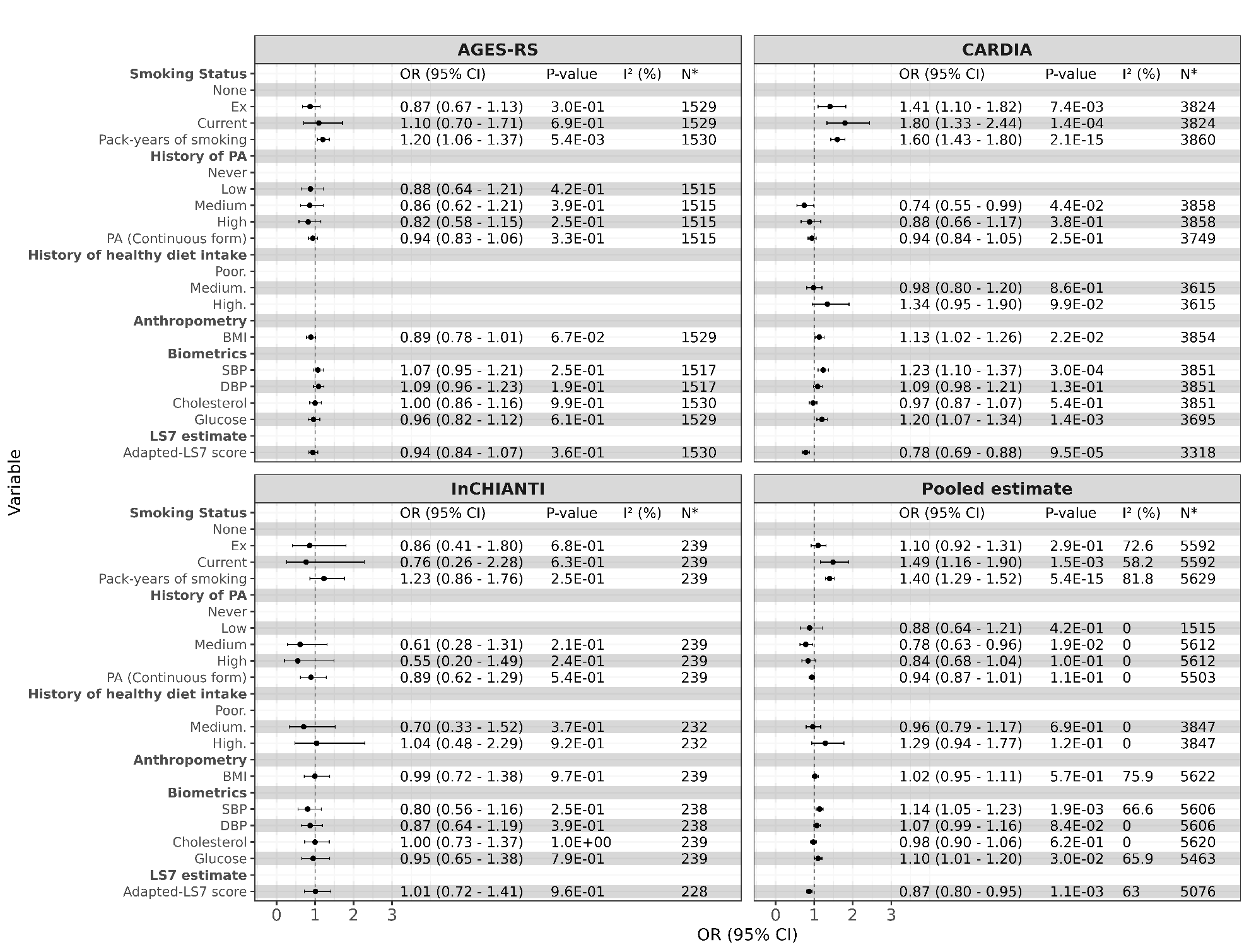
Supplementary Figure 6. The odds ratios (ORs, 95% CIs) for shifting to an accelerated pace of ageing compared to average ageing in the AGE-RS, CARDIA, InCHIANTI, and meta-analysis of the three cohorts in 5-year interval.**

All ***p-values*** were derived from two-sided logistic regression analyses, and the p-values reported for the meta-analysis represent pooled p-value estimates across the three cohorts. All models were adjusted for sex, baseline chronological age, white blood cell composition, baseline educational level, baseline DunedinPACE, and batch effects. Additionally, we adjusted the BP, cholesterol, and glucose models for the use of relevant medications at baseline. Models for the CARDIA cohort were further adjusted for race and data collection centre.

PA: Physical activity; SBP: Systolic Blood Pressure; DBP: Diastolic Blood Pressure; BMI: Body Mass Index; Adapted-LS7: Adapted Life’s Simple 7. Please refer to Supplementary File 2 for definitions of PA.

N* is the number of observations in each model; in meta-analyses, N* exceeds the number of unique samples.
