# Supplementary material for: Mid- and late-life cardiovascular health indicators and changes in biological ageing Markers; A multi-cohort study
Source: eBioMedicine. 2025 Nov 11;122:106016. doi: 10.1016/j.ebiom.2025.106016 (PMC12657379; doi:10.1016/j.ebiom.2025.106016)
Supplement: Supplementary File 1 [file mmc10.docx]

**Supplementary File 1: Description of study cohorts**

This study draws on data from three large, well-characterized, population-based cohorts conducted in Iceland, the United States, and Italy. These cohorts were established to investigate cardiovascular, metabolic, and aging-related health outcomes in community-dwelling adults, and they provide extensive longitudinal and cross-sectional information. Because the sample sizes were determined by the existing cohorts rather than by prospective recruitment, a formal power calculation was not applicable. Below, we provide a detailed description of each cohort, including study design, recruitment strategies, data collection methods, and relevant characteristics of the participants.

### Age, Gene/Environment Susceptibility-Reykjavik Study (AGES-RS)

AGES-RS is a population-based longitudinal cohort, which originated from the Icelandic Heart Association’s Reykjavik Study-RS, (1967 to 1996).^1^ The aim of the founding Reykjavik Study (1967 – 1997) cohort was to study cardiovascular disease and risk factors in middle-aged individuals living in Reykjavik, Iceland. The cohort included all people living in Reykjavik at the start of the study and were born 1907-1935. The AGES-RS cohort (n=5,764, collected between 2002 and 2006) was a follow-up of surviving RS individuals and aimed to investigate the interplay between genetic and environmental factors and their impact on various common age-related conditions. Approximately 5 years later, 3,316 participants underwent a second assessment, conducted between 2007 and 2011.

Whole blood-derived DNA samples were processed at the Huge-F facility (<http://glimdna.org/>) (Accessed on Jun 26^th^, 2023) at Erasmus Medical Center, Rotterdam, the Netherlands. The samples were bisulfite treated using the Zymo EZ-96 DNA Methylation Kit (Zymo Research, Irvine, CA, USA), and DNA methylation levels were analyzed using Illumina's Infinium MethylEPIC V1 Manifest B5 platform (Illumina Inc., San Diego, CA), covering 865,918 CpGs. The BeadChip intensity data were then processed to obtain β-values. The raw beta values underwent Quantile Normalization. We conducted quality control (QC) analysis on the DNA methylation data, excluding the following after QC: i) probes with a detection p-value > 0.01 in more than 10% of the samples (n=1,851), ii) individuals with a probe detection p-value ≥ 0.01 (n=9), iii) all non-CpG probes (n=22,525), iv) probes with a low number of beads (n < 3 beads) (n=1,258), and v) cross-reactive probes (n=43,254). After QC, we retained 819,802 probes from autosomal chromosomes for the final analysis. After quality control, 2,602 participants were included at baseline, of whom 2,081 also completed the second assessment and were included in the longitudinal analysis.

We collected smoking status using self-reported questionnaire (never, former, and current). To address recall bias in reported smoking history and to account for the effect of passive smoking on DNA methylation, we also estimated pack-years of smoking based on DNA methylation, as described by Lu et al.^2^ Physical activity levels were assessed through questionnaire responses indicating hours per week of moderate-to-vigorous PA over the past 12 months. History of moderate-to-vigorous PA was categorized as follows: Never: (0 hrs/week); Low: (> 0 and <1.5 hrs/week); Moderate (≥ 1.5 and < 5.5 hrs/week); and High (≥ 5.5 hrs/week). BMI was derived from measured weight and height (kg/m²). Total cholesterol and fasting glucose levels were both analyzed using comparable enzymatic procedures on a chemistry analyzer (Hitachi 912, Roche Diagnostics, Switzerland, 1999). Systolic (SBP) and diastolic (DBP) levels were calculated as the average of two readings taken with a mercury sphygmomanometer following a 5-minute rest between measurements. These cardiovascular-related risk factors were also assessed with similar methods during participants' midlife (mean age of 50 [SD 6.2] years).^1^

Of the 2,602 baseline participants, 2,081 individuals had DNA methylation data at follow-up, while 20% (n = 521) did not attend the follow-up assessment. The proportion of females was similar between those who dropped out and those who attended follow-up assessment (57.2% vs. 57.6%). However, individuals who did not attend the follow-up were significantly older at baseline (mean age = 79.6 years, SD = 5.2) compared to those who did (mean age = 75.5 years, SD = 4.8; P < 0.05). They also had a higher DunedinPACE score (mean = 1.11, SD = 0.12 vs. 1.09, SD = 0.11; P < 0.05).

### The Coronary Artery Risk Development in Young Adults (CARDIA)

The Coronary Artery Risk Development in Young Adults (CARDIA) study is a prospective cohort study aimed at identifying risk factors influencing the development of cardiovascular diseases, including coronary artery disease, in young adults. Data were collected from four U.S. urban areas: Chicago, Illinois; Birmingham, Alabama; Oakland, California; and Minneapolis, Minnesota. Established in 1984, CARDIA received approval from the institutional review boards of the corresponding study centers, and all participants provided written informed consent at the time of the examination. The initial study design ensured equal representation across key demographic groups: Black and White participants, men and women, age groups (18–24 vs. 25–30 years), and educational levels (high school graduate or less vs. more than high school).^3,4^ At baseline (Y0), the CARDIA study included 5,115 Black and White men and women aged 18–30 years. Data have since been collected at multiple time points, including year 2 (Y2), Y5, Y7, Y10, Y15, Y20, Y25, and Y30, and with recently completed Y35.^5^

Genome-wide DNA methylation profiling for CARDIA samples, in the TOPMed program, was processed using the Infinium MethylationEPIC BeadChip. Data processing was done in R using the minfi^6^ and ENmix^7^ packages. Quality control excluded CpG sites with a detection rate < 95% and samples with > 5% low-quality measurements or low bisulfite conversion probe intensity. Data preprocessing was performed using preprocessNoob and β-values were normalized using the minfi package. This dataset was used for epigenetic age calculations.

After removing outliers, the number of participants were: Y15: 1,568; Y20: 1,807; Y25: 2,715; and Y30: 2,597. Among them, 1,394 had epigenetic clock data for both Y15 and Y20, 1,697 for both Y20 and Y25, 2,467 for both Y25 and Y30, and 1,393 for both Y15 and Y30. DunedinPACE scores were estimated using published algorithms^8^ and R (version 4.3.0) with publicly available code from GitHub (<https://github.com/danbelsky/DunedinPACE/>).

CARDIA participants also completed structured health-related questionnaires to provide information on sociodemographic characteristics and lifestyle factors, including physical activity history, diet and smoking status. Smoking status was categorized as "non-smoker," "former smoker," or "current smoker" based on self-reported responses. Physical activity history was assessed using self-reported data on various types of activity over the past 12 months, including vigorous activities (e.g., jogging and racket sports), leisure activities, and work-related activities. These data were used to calculate total physical activity intensity scores, which estimate the number of kilocalories expended per activity and were expressed in exercise units.^9^

In addition to questionnaire data, clinical measurements and biometric variables were collected, including blood pressure, lipids, fasting blood glucose, weight, and height. Participants were asked to fast for at least 12 h before each examination and to avoid smoking or engaging in heavy physical activity for at least 2 hours before blood draw.^10,11^ CARDIA’s Life’s Simple 7 (LS7) scores were estimated according to a previously published approach.^5^

### InCHIANTI study

The InCHIANTI study is a prospective cohort designed to investigate biological and functional changes associated with aging in the Chianti region of Italy. Detailed descriptions of the study design and data collection protocols are available elsewhere.^12,13^ Briefly, the study received ethical approval from the *Istituto Nazionale Riposo e Cura Anziani* (INRCA) Institutional Review Board in Italy. All participants provided written informed consent prior to their enrollment in the study. Data collection included socio-demographic characteristics, clinical measurements, and biomarkers from blood samples, with assessments conducted at three time points (1998, 2007, and 2013). In the present analysis, there were 678 participants at baseline, and of these, 637 had DNA methylation data available for both 1998 and 2007, 344 had data for both 2007 and 2013, and 364 had data for both 1998 and 2013.^14^

DNA samples were extracted from whole blood, bisulfite-converted using Zymo EZ-96 DNA Methylation Kit (Zymo Research Corp., Irvine, CA), and analyzed for DNA methylation using the Illumina Infinium HumanMethylation450 BeadChip.^14^ Comprehensive quality control and normalization processes were performed on the DNA methylation data before deriving epigenetic clock estimates. Quality control was performed using “minfi”^6^, and “SeSAMe”^15^ R packages, and have been detailed previously. Briefly, the steps included background correction, multidimensional scaling, and verifying the consistency of estimated sex chromosome copy numbers with self-reported sex. The call rate threshold for sample inclusion was > 95%. Probes with bead count <3 in ≥5% of samples were removed. Additionally, samples and probes with ≥5% detection p-values >.01 were excluded. After the quality control, 678 participants from 1998, 658 from 2007, and 385 from 2013, and 429,527 probes remained. Individual methylation values were reported as beta values ranging from 0 (no methylation) to 1 (complete methylation).

DunedinPACE scores were calculated based on the steps presented by Belsky et al article,^8^ using the DundedinPACE R packages (<https://github.com/danbelsky/DunedinPACE>) while the PCHorvath1, PCHannum, PCPhenoAge, and PCGrimAge were calculated using the web-based calculator (https://horvath.genetics.ucla.edu/html/dnamage/) on May 31, 2024.

Information on sex, age, smoking status, physical activity, food intake, and medication use history was collected using a structured questionnaire. Food intake was collected using a validated food frequency questionnaire (FFQ) questionnaire.^16^  During clinic study visits, participants' weight and height was measured and body mass index (BMI) was calculated as weight in kilograms divided by the square of height in meters (kg/m²). Health professionals measured blood pressure (BP) three times, at two-minute intervals, using a standard mercury sphygmomanometer while participants rested in the supine position. The mean of the last two BP readings were considered as the final BP for each participant.

Participants were instructed to fast for at least eight hours before blood sample collection. Blood tests were performed to measure fasting plasma glucose and total cholesterol levels. Trained staff determined fasting plasma glucose using an enzymatic colorimetric assay, specifically a modified glucose oxidase-peroxidase method, with a Roche-Hitachi 917 analyzer (Roche Diagnostics, GmbH, Mannheim, Germany), and total cholesterol using commercial enzymatic tests from Roche Diagnostics.^16^ The Life’s Simple 7 (LS7) score was calculated according to previously published method.^16^

Drop out between the exams varied. The dropout rate between 1998 and 2007 was 6.04%, ( n = 41/678), it was higher between 2007 and 2013 (47.7%, n = 314/658). Participants who dropped out in 2013 had a higher pace of aging (mean (SD) DunedinPACE: 1.13 (0.13) vs. 1.08 (0.13), P < 0.05), were older (mean (SD): 76.78 (13.99) vs. 67.65 (15.62), P < 0.05), and had a higher cumulative smoking exposure (mean (SD) pack-years: 10.74 (8.08) vs. 7.62 (8.40), P < 0.05).

# Reference

1. Harris TB, Launer LJ, Eiriksdottir G, et al. Age, Gene/Environment Susceptibility-Reykjavik Study: multidisciplinary applied phenomics. *Am J Epidemiol*. 2007;165(9):1076-1087. doi:10.1093/aje/kwk115

2. Lu AT, Quach A, Wilson JG, et al. DNA methylation GrimAge strongly predicts lifespan and healthspan. *Aging (Albany NY)*. 2019;11(2):303-327. doi:10.18632/aging.101684

3. Friedman GD, Cutter GR, Donahue RP, et al. CARDIA: study design, recruitment, and some characteristics of the examined subjects. *J Clin Epidemiol*. 1988;41(11):1105-1116. doi:10.1016/0895-4356(88)90080-7

4. Joyce BT, Gao T, Zheng Y, et al. Epigenetic age acceleration reflects long-term cardiovascular health. *Circ Res*. 2021;129(8):770-781. doi:10.1161/CIRCRESAHA.121.318965

5. Forrester SN, Baek J, Hou L, Roger V, Kiefe CI. A comparison of 5 measures of accelerated biological aging and their association with incident cardiovascular disease: The CARDIA study. *J Am Heart Assoc*. 2024;13(8):e032847. doi:10.1161/JAHA.123.032847

6. Aryee MJ, Jaffe AE, Corrada-Bravo H, et al. Minfi: a flexible and comprehensive Bioconductor package for the analysis of Infinium DNA methylation microarrays. *Bioinformatics*. 2014;30(10):1363-1369. doi:10.1093/bioinformatics/btu049

7. Xu Z, Niu L, Li L, Taylor JA. ENmix: a novel background correction method for Illumina HumanMethylation450 BeadChip. *Nucleic Acids Res*. 2016;44(3):e20. doi:10.1093/nar/gkv907

8. Belsky DW, Caspi A, Corcoran DL, et al. DunedinPACE, a DNA methylation biomarker of the pace of aging. *Elife*. 2022;11. doi:10.7554/elife.73420

9. Dougherty RJ, Moonen J, Yaffe K, et al. Smoking mediates the relationship between SES and brain volume: The CARDIA study. *PLoS One*. 2020;15(9):e0239548. doi:10.1371/journal.pone.0239548

10. Reis JP, Allen NB, Bancks MP, et al. Duration of diabetes and prediabetes during adulthood and subclinical atherosclerosis and cardiac dysfunction in middle age: The CARDIA Study. *Diabetes Care*. 2018;41(4):731-738. doi:10.2337/dc17-2233

11. Correction: Association of smoking and right ventricular function in middle age: CARDIA study. *Open Heart*. 2020;7(1):e001270corr1. doi:10.1136/openhrt-2020-001270corr1

12. Ferrucci L, Bandinelli S, Benvenuti E, et al. Subsystems contributing to the decline in ability to walk: Bridging the gap between epidemiology and geriatric practice in the InCHIANTI study. *J Am Geriatr Soc*. 2000;48(12):1618-1625. doi:10.1111/j.1532-5415.2000.tb03873.x

13. Tharakan R, Ubaida-Mohien C, Moore AZ, Hernandez D, Tanaka T, Ferrucci L. Blood DNA methylation and aging: A cross-sectional analysis and longitudinal validation in the InCHIANTI study. *J Gerontol A Biol Sci Med Sci*. 2020;75(11):2051-2055. doi:10.1093/gerona/glaa052

14. Kuo PL, Moore AZ, Tanaka T, et al. Longitudinal changes in epigenetic clocks predict survival in the InCHIANTI cohort. *medRxiv*. Published online September 15, 2024. doi:10.1101/2024.09.13.24313620

15. Zhou W, Triche TJ Jr, Laird PW, Shen H. SeSAMe: reducing artifactual detection of DNA methylation by Infinium BeadChips in genomic deletions. *Nucleic Acids Res*. 2018;46(20):e123. doi:10.1093/nar/gky691

16. Jin Y, Tanaka T, Ma Y, Bandinelli S, Ferrucci L, Talegawkar SA. Cardiovascular health is associated with disability among older community dwelling men and women. *J Aging Health*. 2019;31(8):1339-1352. doi:10.1177/0898264318778417
