# Supplementary material for: Mid- and late-life cardiovascular health indicators and changes in biological ageing Markers; A multi-cohort study
Source: eBioMedicine. 2025 Nov 11;122:106016. doi: 10.1016/j.ebiom.2025.106016 (PMC12657379; doi:10.1016/j.ebiom.2025.106016)
Supplement: Supplementary File 2 [file mmc11.docx]

**Supplementary File 2: Definition of physical activity scores across the three cohorts**

## AGES-RS

Physical activity (PA) levels were assessed using questionnaire responses reporting hours per week of moderate-to-vigorous PA over the past 12 months.^1,2^ The continuous form of PA variable reflected total hours per week and was standardized into SD units, while the categorical variable was defined as follows: Never (0 hrs/week), Low (>0 to <1.5 hrs/week), Moderate (≥1.5 to <5.5 hrs/week), and High (≥5.5 hrs/week).

## InCHIANTI

In the InCHIANTI cohort, PA intensity was assessed through a questionnaire^3^ with responses ranging from 1 to 7: (1) Hardly any activity, (2) Mostly sitting/some walking, (3) Light exercise (2–4 hrs/week), (4) Moderate (1–2 hrs or light >4 hrs/week), (5) Moderate exercise (>3 hrs/week), (6) Intense exercise several times/week, and (7) Walking ≥5 km/day, ≥5 days/week for ≥5 years. Based on these responses, we derived a categorical PA variable: responses 1–2 as "Never or Low," 3 as "Medium," and 4 or higher as "High." The PA intensity variable ranged from 1 to 7 scores and was converted into SD units before analysis.

## CARDIA

Physical activity history was assessed using self-reported data on various types of activity over the past 12 months, including vigorous activities (e.g., jogging and racket sports), leisure activities, and work-related activities. These data were used to calculate total physical activity intensity scores, which estimate the number of kilocalories expended per activity^4^ and were expressed in exercise units.^5^ In this study, continues form of PA was the total physical activity intensity scores, ranged from 0 to 2,184 and was converted to SD units. The categorical form of PA in the CARDIA cohort were based on the LS7 scores for PA, 0=Poor (Never or Low)), 1=Medium, and 2= Ideal (High), as reported in the previous study.^6^

# References:

1. Sedaghat S, Lutsey PL, Ji Y, et al. Association of change in cardiovascular risk factors with incident dementia. *Alzheimers Dement*. 2023;19(5):1821-1831. doi:10.1002/alz.12818

2. Harris TB, Launer LJ, Eiriksdottir G, et al. Age, Gene/Environment Susceptibility-Reykjavik Study: multidisciplinary applied phenomics. *Am J Epidemiol*. 2007;165(9):1076-1087. doi:10.1093/aje/kwk115

3. Patel KV, Coppin AK, Manini TM, et al. Midlife physical activity and mobility in older age. *Am J Prev Med*. 2006;31(3):217-224. doi:10.1016/j.amepre.2006.05.005

4. Dougherty RJ, Moonen J, Yaffe K, et al. Smoking mediates the relationship between SES and brain volume: The CARDIA study. *PLoS One*. 2020;15(9):e0239548. doi:10.1371/journal.pone.0239548

5. Jacobs DR Jr, Hahn LP, Haskell WL, Pirie P, Sidney S. Validity and reliability of short physical activity history: Cardia and the Minnesota heart health program. *J Cardiopulm Rehabil*. 1989;9(11):448-459. doi:10.1097/00008483-198911000-00003

6. Joyce BT, Gao T, Zheng Y, et al. Epigenetic age acceleration reflects long-term cardiovascular health. *Circ Res*. 2021;129(8):770-781. doi:10.1161/CIRCRESAHA.121.318965
