# Supplementary material for: Mid- and late-life cardiovascular health indicators and changes in biological ageing Markers; A multi-cohort study
Source: eBioMedicine. 2025 Nov 11;122:106016. doi: 10.1016/j.ebiom.2025.106016 (PMC12657379; doi:10.1016/j.ebiom.2025.106016)
Supplement: Supplementary File 3 [file mmc12.docx]

**Supplementary File 3: Statistical Equations**

## Prospective analysis

We performed linear mixed models to evaluate the longitudinal association between DunedinPACE (DDPACE) scores and cardiovascular risk factors across the three cohorts (Equation 1). The DDPACE aging score, treated as a continuous variable, served as the outcome, with “time-varying” lifestyle factors from both baseline and follow-up time points as the predictors. We adjusted models for the effects of sex, chronological age, educational levels, white blood cell proportions, and assay batch. Additionally, beyond adjusting for these covariates, we incorporated the effects of antihypertensive, lipid-lowering, and glucose-lowering medications when modeling BP, total cholesterol, and fasting plasma glucose, respectively. Furthermore, models for the CARDIA cohort were further adjusted for race and data collection center.

$\boldsymbol{Y}_{\boldsymbol{ij}}\boldsymbol{=}\boldsymbol{\beta}_{\boldsymbol{0}}\boldsymbol{+}\boldsymbol{\beta}_{\boldsymbol{1}}\boldsymbol{X}_{\boldsymbol{1}\boldsymbol{ij}}\boldsymbol{+}\boldsymbol{\beta}_{\boldsymbol{2}}\boldsymbol{X}_{\boldsymbol{2}\boldsymbol{ij}}\boldsymbol{+\ldots+}\boldsymbol{\beta}_{\boldsymbol{p}}\boldsymbol{X}_{\boldsymbol{pij}}\boldsymbol{+}\boldsymbol{u}_{\boldsymbol{i}}\boldsymbol{+}\boldsymbol{\epsilon}_{\boldsymbol{ij}}\boldsymbol{Equation 1}$

*Where, Y_ij_​: The DDPACE score for individual i (i= 1,2, 3, ..n) at time j (j=1,2,3,..m); β_0_​: Fixed effect of the intercept; β_1_,β_2_,…,β_p:_ Fixed effects of the predictor (β_1_) and covariates (β_2_ to β_p_);* *X_1ij_​: time variant exposure variable (e.g. smoking status), X_2ij_​ to X_pij_​: covariates (e.g. sex and educational level); u_i​_: Random effect for the individual i; ϵ_ij_​: Residual error term for individual i at time j.*

Furthermore, to explore the long-term association of the CV risk factor on late-age in the AGES-RS cohort, we examined the prospective association of mid-life risk factors (measured at mean age 50 years) to the baseline AGES-RS DDPACE of aging (measured at mean age 76 yrs, n=2,602) (Equation 2). The models were adjusted for sex, baseline chronological age, baseline education level, white blood cell composition, and assay batch. For models examining BP, total cholesterol, and fasting plasma glucose, we additionally accounted for the use of antihypertensive, lipid-lowering, and glucose-lowering medications, respectively.

$Y\boldsymbol{=}\boldsymbol{\beta}_{\boldsymbol{0}}\boldsymbol{+}\boldsymbol{\beta}_{\boldsymbol{1}}\boldsymbol{X}_{\boldsymbol{1}}\boldsymbol{+}\boldsymbol{\beta}_{\boldsymbol{2}}\boldsymbol{X}_{\boldsymbol{2}}\boldsymbol{+\ldots+}\boldsymbol{\beta}_{\boldsymbol{k}}\boldsymbol{X}_{\boldsymbol{k}}\boldsymbol{+\epsilon Equation 2}$

*Where, Y is DDPACE score at Time 1; β_0_: the intercept term; β_1_, β_2_, …, β_k_ are coefficients for the predictor X_1_ (e.g. baseline smoking) and covariates of X_2_,…,X_k_​ (e.g. sex and batch); and ε: error term.*

## Change analysis

We calculated the change in DDPACE scores (Time 2 – Time 1) and employed linear regression models (Equation 3) to examine the associations between this change (outcome) and baseline cardiovascular risk factors (exposures) across the three cohorts. We controlled for sex, baseline age, baseline DDPACE scores, baseline educational attainment, proportions of white blood cells, and assay batch across all models. BP, total cholesterol, and fasting glucose models, we also considered relevant medication use, including antihypertensive, lipid-lowering, and glucose-lowering drugs. Analyses using CARDIA data included additional adjustments for participants' race and study site.

$Y\boldsymbol{=}\boldsymbol{\beta}_{\boldsymbol{0}}\boldsymbol{+}\boldsymbol{\beta}_{\boldsymbol{1}}\boldsymbol{X}_{\boldsymbol{1}}\boldsymbol{+}\boldsymbol{\beta}_{\boldsymbol{2}}\boldsymbol{X}_{\boldsymbol{2}}\boldsymbol{+\ldots+}\boldsymbol{\beta}_{\boldsymbol{k}}\boldsymbol{X}_{\boldsymbol{k}}\boldsymbol{+\epsilon Equation 3}$

*Where, Y is the difference between DDPACE score at Time 2 and Time 1 (Time 2 – Time 1); β_0_: the intercept term; β_1_, β_2_, …, β_k_ are coefficients for the predictor X_1_ (e.g. baseline smoking) and covariates of X_2_,…,X_k_​ (e.g. sex and batch); and ε: error term.*

## Shift analyses

Participants were classified into "Slow," "Average," and "Fast" agers at two time points using mean ± SD DDPACE thresholds (with Time 1 thresholds applied consistently across both time points). Participants were then reclassified as Decelerators, Average agers, or Accelerators based on their baseline and follow-up categories (Please see Methods section of the manuscript for details). Subsequently, logistic regression analyses were performed across the three cohorts to assess the relationships using these categories as the outcome and the cardiovascular-related risk factors as exposures (Equation 4). Our shift analysis focused on individuals who shifted to a fast or slow DDPACE of aging during the follow-up period, while those with a consistently ‘Fast’ or ‘Slow’ DDPACE of aging were excluded. We adjusted the models for sex, baseline chronological age, baseline education level, white blood cell proportions, baseline DDPACE aging pace, and batch effects. Furthermore, when modeling BP, total cholesterol, and fasting plasma glucose, we included the effects of antihypertensive, lipid-lowering, and glucose-lowering medications, respectively, in addition to the other covariates. Analyses using CARDIA data included additional adjustments for participants' race and study site.

$\log\left( \frac{P\left( Y=1 \right)}{1-P\left( Y=1 \right)} \right)\boldsymbol{=}\boldsymbol{\beta}_{\boldsymbol{0}}\boldsymbol{+}\boldsymbol{\beta}_{\boldsymbol{1}}\boldsymbol{X}_{\boldsymbol{1}}\boldsymbol{+}\boldsymbol{\beta}_{\boldsymbol{2}}\boldsymbol{X}_{\boldsymbol{2}}\boldsymbol{+\ldots+}\boldsymbol{\beta}_{\boldsymbol{k}}\boldsymbol{X}_{\boldsymbol{k}}\boldsymbol{+\epsilon Equation 4}$

*Where, P (Y=1) is the probability of shifting to an Accelerated pace of aging compared to Decelerated pace.*

*log(P(Y=1)/1-P(Y=1)) is the log odds; β_0_: the intercept term; β_1_, β_2_, …, β_k_ are coefficients for the predictor X_1_ (e.g. baseline smoking) and covariates of X_2_,…,X_k_​ (e.g. sex and batch); and ε: error term.*
