# Supplementary material for: Mid- and late-life cardiovascular health indicators and changes in biological ageing Markers; A multi-cohort study
Source: eBioMedicine. 2025 Nov 11;122:106016. doi: 10.1016/j.ebiom.2025.106016 (PMC12657379; doi:10.1016/j.ebiom.2025.106016)
Supplement: Supplementary Table 1 [file mmc13.docx]

**Supplementary Table 1. Participant characteristics at the baseline and follow-up assessments across the three cohorts**

|  | **Study** | | | | | | | | |
| --- | --- | --- | --- | --- | --- | --- | --- | --- | --- |
|  | **AGES-RS** | | **InCHIANTI** | | | **CARDIA** | | | |
| **Characteristics** | **AGES-RS (2006)** | **AGES-RS (2011)** | **InCHIANTI (1998)** | **InCHIANTI (2007)** | **InCHIANTI (2013)** | **CARDIA (Y15)** | **CARDIA (Y20)** | **CARDIA (Y25)** | **CARDIA (Y30)** |
| Total number of participants, N | **2,602** | **2,081** | **678** | **658** | **385** | **1,568** | **1,807** | **2,715** | **2,597** |
| Sex (women, %) | 1,496 (57.5) | 1,202 (57.6) | 377 (55.6) | 362 (55) | 216 (56.1) | 1,030 (65.6) | 1,104 (61.1) | 1,578 (58.1) | 1,506 (58) |
| Chronological age (years), mean (SD) | 76.32 (5.2) | 80.72 (4.9) | 62.9 (15.7) | 72 (15.5) | 73.4 (15.8) | 40.3 (3.6) | 45.7 (3.6) | 50.8 (3.6) | 55.8 (3.6) |
| Follow-up years, median (IQR) | NA | 5.15 (0.28) | NA | 9.0 (0.01) | 5.0 (1.0) | NA | 4.98 (0.37) | 4.99 (0.38) | 5.02 (0.34) |
| **Race, n (%)**^β^ |  |  |  |  |  |  |  |  |  |
| White, not Hispanic | - | - | - | - | - | 864 (55.1) | 983 (54.4) | 1515 (55.8) | 1439 (55.4) |
| Black, not Hispanic | - | - | - | - | - | 702 (44.7) | 821 (45.4) | 1190 (43.8) | 1149 (44.2) |
| Others | - | - | - | - | - | 3 (0.2) | 3 (0.2) | 9 (0.3) | 8 (0.3) |
| **Pace of biological age** |  |  |  |  |  |  |  |  |  |
| DunedinPACE score, mean (SD) | 1.10 (0.11) | 1.13 (0.12) | 1.06 (0.12) | 1.1 (0.13) | 1.09 (0.13) | 0.92 (0.13) | 0.93 (0.13) | 0.95 (0.13) | 0.97 (0.14) |
| **Modifiable behaviors** |  |  |  |  |  |  |  |  |  |
| **smoking status (n (%))** |  |  |  |  |  |  |  |  |  |
| Never-smokers | 1,134 (43.6) | 851 (40.8) | 384 (56.6) | 362 (55) | 206 (53.5) | 952 (60.7) | 1,123 (62.1) | 1,664 (61.3) | 1,608 (61.9) |
| Former smokers | 1,167 (44.9) | 1,010 (48.4) | 159 (23.5) | 227 (34.5) | 144 (37.4) | 309 (19.7) | 363 (20.1) | 596 (22) | 595 (22.9) |
| Current smokers | 296 (11.4) | 181 (8.7) | 135 (19.9) | 69 (10.5) | 35 (9.1) | 305 (19.4) | 310 (17.2) | 417 (15.4) | 316 (12.2) |
| Pack-years of smoking, mean (SD)^*^ | 15.44 (7.4) | 15.40 (7.0) | 8.52 (8.7) | 9.04 (8.4) | 6.79 (8.5) | 7.45 (9.4) | 8.44 (9.46) | 9.47 (9.3) | 10.28 (9.0) |
| **Moderate-to-vigorous PA** |  |  |  |  |  |  |  |  |  |
| Never | 1,084 (41.70) | 1,015 (48.7) | NA | NA | NA | NA | NA | NA | NA |
| Never or low | NA | NA | 60 (8.8) | 269 (40.9) | 141 (36.6) | 289 (18.4) | 362 (20) | 525 (19.3) | 529 (20.4) |
| Low | 616 (23.7) | 197 (9.4) | NA | NA | NA | NA | NA | NA | NA |
| Moderate | 447 (17.2) | 436 (20.9) | 275 (40.6) | 263 (40) | 125 (32.5) | 535 (34.1) | 604 (33.4) | 916 (33.7) | 885 (34.1) |
| High | 420 (16.1) | 392 (18.8) | 339 (50) | 126 (19.1) | 119 (30.9) | 740 (47.2) | 833 (46.1) | 1270 (46.8) | 1126 (43.4) |
| PA total intensity score in Z-scores, median (IQR) ^¥^ | -0.6 (0.6) | -0.6 (0.6) | 0.8 (0.9) | -0.14 (0.9) | -0.14 (1.9) | -0.2 (1.3) | -0.2 (1.5) | -0.2 (1.5) | -0.3 (1.4) |
| **Biometrics** |  |  |  |  |  |  |  |  |  |
| BMI (kg/m^2^), mean (SD) | 26.98 (4.3) | 26.74 (4.4) | 27.18 (3.9) | 27.02 (4.2) | 26.9 (4.3) | 28.71 (7.11) | 29.37 (7.0) | 30.04 (7.1) | 30.43 (7.2) |
| SBP (mmHg), median (IQR) | 139 (24.3) | 142 (28) | 140 (28.8) | 130 (22.5) | 130 (22.5) | 110 (17) | 114 (18) | 117 (19) | 118 (20) |
| DBP (mmHg), median (IQR) | 73 (12) | 70 (16) | 80 (10) | 80 (12.5) | 72.5 (10) | 73 (14) | 72 (14) | 73 (14) | 73 (14) |
| Fasting plasma glucose (mg/dL), median (IQR) | 99.1 (12.6) | 98.2 (13.5) | 88 (15) | 89 (19) | 93 (16.3) | 84 (11) | 93 (11) | 93 (13) | 95 (14) |
| Total cholesterol (mg/dL), mean (SD) | 217.48 (44.3) | 202.47 (44.7) | 216.13 (39.8) | 205.17 (38.6) | 213.26 (43.1) | 183.68 (34.57) | 184.49 (33.6) | 192.19 (36.8) | 191.29 (38) |
| **Medication history** |  |  |  |  |  |  |  |  |  |
| Taking any antihypertensive medication? (Yes, n (%)) | 1,212 (46.6) | 1,509 (72.4) | 211 (31.1) | 326 (49.5) | 205 (53.2) | 114 (7.3) | 311 (17.2) | 714 (26.3) | 846 (32.6) |
| Using any lipid-lowering agents? (Yes, n (%)) | 615 (23.6) | 794 (38.1) | 31 (4.6) | 85 (12.9) | 85 (22.1) | 35 (2.2) | 167 (9.2) | 434 (16) | 529 (20.4) |
| Taking oral hypoglycemic agents? (Yes, n (%)) | 151 (5.8) | 149 (7.1) | 34 (5) | 68 (10.3) | 42 (10.9) | 37 (2.4) | 85 (4.7) | 207 (7.6) | 287 (11.1) |
| Adapted-LS7, median (IQR)**^♀^** | 7 (3) | 7 (2) | 6 (3) | 7 (2) | 7 (3) | 9 (3) | 9 (3) | 9 (3) | 9 (3) |

^β^The AGES-RS and InCHIANTI study populations are consisted of White, non-Hispanic individuals.

^*^*Pack-years of smoking were estimated using a DNA methylation metric,* Lu AT et al. *Aging (Albany NY)*. 2019;11(2):303-327

*^¥^ See supplementary File 2 for definition of PA in each cohort.*

**^♀^**Due to a lack of or insufficient diet data, the Adapted-LS7 scores for the AGES-RS cohort were estimated based on six cardiovascular-related factors, with a maximum achievable score of 12. In contrast, for the CARDIA and InCHIANTI cohorts, LS7 scores were based on a total of 14 scores.

*PA: Physical activity; SBP: Systolic Blood Pressure; DBP: Diastolic Blood Pressure; BMI: Body Mass Index*
