# Supplementary material for: Mid- and late-life cardiovascular health indicators and changes in biological ageing Markers; A multi-cohort study
Source: eBioMedicine. 2025 Nov 11;122:106016. doi: 10.1016/j.ebiom.2025.106016 (PMC12657379; doi:10.1016/j.ebiom.2025.106016)
Supplement: Supplementary Table 2 [file mmc14.docx]

**Supplementary table 2. Distribution of cardiovascular characteristics by shift in DunedinPACE categories from baseline to first follow-up across the three cohorts.**

|  | AGES-RS (2006 vs. 2011, 2,081) | | | | CARDIA (Y15 vs. Y20, n=1,395) | | | | InCHIANTI (1998 vs. 2007, n=637) | | | |
| --- | --- | --- | --- | --- | --- | --- | --- | --- | --- | --- | --- | --- |
|  | **Decelerators (n=276)** | **Average agers (n=1,136)** | **Accelerators (n=669)** | **P** | **Decelerators (n=237)** | **Average agers (n=789)** | **Accelerators (n=369)** | **P** | **Decelerators (n=92)** | **Average agers (n=319)** | **Accelerators (n=226)** | **P** |
| *Smoking status (n (%))* | |  |  |  |  |  |  |  |  |  |  |  |
| Never-smokers, n (%) | *148 (53.6%)* | *499 (43.9%)* | *248 (37.1%)* | 8.94e-02 | 157 (66.2%) | 505 (64%) | 191 (51.8%) | 5.18e-06 | 59 (64.1%) | 183 (57.4%) | 117 (51.8%) | ns |
| Ex-smokers, n (%) | *105 (38%)* | *534 (47%)* | *313 (46.8%)* |  | 45 (19%) | 156 (19.8%) | 73 (19.8%) |  | 16 (17.4%) | 74 (23.2%) | 62 (27.4%) |  |
| Current-smokers, n (%) | *23 (8.3%)* | *100 (8.8%)* | *106 (15.8%)* |  | 35 (14.8%) | 126 (16%) | 104 (28.2%) |  | 17 (18.5%) | 62 (19.4%) | 47 (20.8%) |  |
| Pack-year of smoking, mean (SD)^*^ | 12.41 (6.84) | 14.25 (6.48) | 17.57 (7.99) | 5.25E-30 | 5.13 (8.87) | 6.14 (7.95) | 11.1 (11.02) | 3.67E-20 | 4.03 (9.01) | 8.31 (7.83) | 10.94 (8.73) | 2.40E-10 |
| History of moderate-to-vigorous PA | |  |  |  |  |  |  |  |  |  |  |  |
| None, n (%) | *96 (34.8%)* | *420 (37%)* | *287 (42.9%)* | 1.07e-07 |  |  |  | 5.42e-05 |  |  |  | 0.229 |
| None or low, n (%) |  |  |  |  | 36 (15.2%) | 134 (17%) | 92 (24.9%) |  | 5 (5.4%) | 32 (10%) | 21 (9.3%) |  |
| Low, n (%) | *77 (27.9%)* | *273 (24%)* | *156 (23.3%)* |  |  |  |  |  |  |  |  |  |
| Moderate, n (%) | *57 (20.7%)* | *217 (19.1%)* | *114 (17%)* |  | 63 (26.6%) | 285 (36.1%) | 126 (34.1%) |  | 32 (34.8%) | 124 (38.9%) | 101 (44.7%) |  |
| High, n (%) | *45 (16.3%)* | *212 (18.7%)* | *104 (15.5%)* |  | 138 (58.2%) | 367 (46.5%) | 150 (40.7%) |  | 53 (57.6%) | 162 (50.8%) | 103 (45.6%) |  |
| PA total intensity score in Z scores, median (IQR) *^¥^* | *-0.59 (0.6)* | *-0.59 (0.6)* | *-0.59 (0.6)* | 2.54E-02 | 0.04 (1.4) | -0.22 (1.4) | -0.31 (1.3) | 8.42E-05 | 0.79 (0.94) | 0.79 (0.94) | -0.14 (0.94) | 9.05E-02 |
| BMI (kg/m2), mean (SD) | 26.27 (3.92) | 27.45 (4.17) | 27.32 (4.46) | 1.67E-04 | 26.11 (5.86) | 28.22 (6.15) | 31.11 (8.38) | 1.50E-18 | 26.09 (4.6) | 27.35 (3.8) | 27.49 (3.77) | 1.20E-02 |
| SBP (mmHg), median (IQR) | 136 (28) | 140 (24) | 139 (25) | 1.73E-01 | 109 (17) | 110 (17) | 113 (20) | 2.13E-06 | 130 (30) | 140 (30) | 145 (30) | 6.11E-06 |
| DBP (mmHg), median (IQR) | 72 (11) | 74 (11) | 74 (12) | 1.99E-01 | 71 (14) | 73 (13.5) | 75 (15.25) | 9.21E-05 | 80 (17.5) | 80 (10) | 80 (10) | 4.65E-03 |
| Fasting plasma glucose (mg/dL), median (IQR) | 97.3 (12.61) | 99.1 (12.61) | 100.9 (12.61) | 1.36E-04 | 83 (11) | 83 (11) | 85 (13) | 8.53E-04 | 82 (16.75) | 87 (14) | 89 (17) | 4.45E-03 |
| Total cholesterol (mg/dL), mean (SD) | 220.58 (44.64) | 217.57 (44.18) | 215.41 (43.58) | 2.47E-01 | 180.72 (31.82) | 184.13 (34.54) | 185.97 (37.39) | 1.95E-01 | 205.23 (38.53) | 219.61 (40.5) | 216.51 (40.11) | 1.10E-02 |
| Adapted-LS7, median (IQR)^♀^ | 8 (3) | 7 (2) | 7 (3) | 1.01E-07 | 11 (3) | 10 (3) | 8 (3) | 6.64E-24 | 7 (4) | 6 (3) | 6 (2) | 7.89E-05 |
|  |  |  |  |  |  |  |  |  |  |  |  |  |

Participants were first classified into "Slow," "Average," and "Fast" agers at two time points using mean ± SD DunedinPACE thresholds (with Time 1 thresholds applied consistently across both time points). Participants were reclassified as Decelerators, Average agers, or Accelerators based on their baseline and follow-up categories. PA: Physical activity; SBP: Systolic Blood Pressure; DBP: Diastolic Blood Pressure; BMI: Body Mass Index; adapted-LS7: adapted Life’s Simple 7.

^*^Pack-years of smoking were estimated using a DNA methylation metric, Lu AT et al. *Aging (Albany NY)*. 2019;11(2):303-327

^¥^ Full description of PA variables is provided in Supplementary text 2

***^♀^***Due to a lack of or insufficient diet data, the Adapted-LS7 scores for the AGES-RS and InCHIANTI cohorts were estimated based on six cardiovascular-related factors, with a maximum achievable score of 12. In contrast, for the CARDIA cohort, LS7 scores are based on a total of 14 scores.

**P-values** were generated from **one-way ANOVA** if the data were normally distributed, or from the **Kruskal–Wallis test** if the data were not normally distributed. For categorical variables**, p-values** were generated using **Chi-squared tests**.
