# Supplementary material for: Mid- and late-life cardiovascular health indicators and changes in biological ageing Markers; A multi-cohort study
Source: eBioMedicine. 2025 Nov 11;122:106016. doi: 10.1016/j.ebiom.2025.106016 (PMC12657379; doi:10.1016/j.ebiom.2025.106016)
Supplement: Supplementary Table 3 [file mmc15.docx]

**Supplementary Table 3a. Longitudinal association of cardiovascular-related factors with epigenetic age acceleration (biological ageing - chronological ageing), as estimated by two first generation and two second generation epigenetic clocks, in the AGES-RS cohort.**

| Variable | PC-Horvath |  | PC-Hannum |  | PC-PhenoAge |  | PC-GrimAge |  |
| --- | --- | --- | --- | --- | --- | --- | --- | --- |
|  | **Estimate (95% CI)** | **P-Value** | **Estimate (95% CI)** | **P-Value** | **Estimate (95% CI)** | **P-Value** | **Estimate (95% CI)** | **P-Value** |
| Smoking status |  |  |  |  |  |  |  |  |
| Never | Reference |  |  |  |  |  |  |  |
| Ex | 0.09 (0.03-0.15) | **4.320E-03** | 0.08 (0.02-0.14) | **1.05E-02** | 0.09 (0.03-0.16) | **5.25E-03** | 0.4 (0.35-0.45) | **1.52E-57** |
| Current | 0.12 (0.04-0.19) | **1.498E-03** | 0.11 (0.04-0.18) | **2.59E-03** | 0.08 (0-0.16) | **4.02E-02** | 0.69 (0.63-0.75) | **5.97E-118** |
| Pack-years of smoking | 0.11 (0.08-0.14) | **1.175E-13** | 0.21 (0.19-0.24) | **5.90E-51** | 0.26 (0.23-0.29) | **6.05E-62** | 0.74 (0.73-0.75) | **0.00E+00** |
| History of moderate to Vigorous PA |  |  |  |  |  |  |  |  |
| None | Reference |  |  |  |  |  |  |  |
| Low | -0.02 (-0.04-0.01) | 2.084E-01 | -0.001 (-0.03-0.02) | 7.92E-01 | -0.001 (-0.03-0.03) | 9.08E-01 | -0.001 (-0.02-0.02) | 7.85E-01 |
| Middle | -0.02 (-0.04-0.01) | 2.176E-01 | -0.02 (-0.05-0) | 6.36E-02 | -0.03 (-0.06-0) | **6.71E-02** | -0.02 (-0.04-0) | 8.03E-02 |
| High | -0.03 (-0.06-0) | **3.746E-02** | -0.03 (-0.06-0) | **4.92E-02** | -0.06 (-0.09--0.02) | **1.40E-03** | -0.04 (-0.06--0.02) | **3.04E-04** |
| PA (Continuous form) | -0.01 (-0.02-0) | 5.188E-02 | -0.01 (-0.02-0) | **3.36E-02** | -0.02 (-0.03--0.01) | **3.36E-04** | -0.02 (-0.02--0.01) | **7.81E-05** |
| BMI | -0.01 (-0.03-0.01) | 5.171E-01 | -0.02 (-0.04-0) | **3.94E-02** | -0.01 (-0.03-0.02) | 6.30E-01 | -0.03 (-0.05--0.01) | **6.80E-04** |
| SBP | 0.001 (-0.01-0.01) | 4.332E-01 | 0.001 (-0.01-0.01) | 4.82E-01 | -0.02 (-0.03-0) | **1.37E-02** | -0.01 (-0.02-0) | **2.12E-02** |
| DBP | -0.01 (-0.02-0.01) | 3.418E-01 | 0.001 (-0.01-0.01) | 7.31E-01 | -0.01 (-0.03-0) | 7.95E-02 | -0.01 (-0.02-0) | **1.95E-02** |
| Cholesterol | -0.02 (-0.03-0) | **4.097E-02** | -0.01 (-0.03-0.01) | 2.64E-01 | -0.04 (-0.06--0.02) | **1.49E-04** | -0.01 (-0.03-0) | **4.88E-02** |
| Glucose | 0.001 (-0.01-0.01) | 9.344E-01 | 0.01 (-0.01-0.02) | 3.40E-01 | 0.02 (0-0.04) | **3.96E-02** | 0.01 (0-0.02) | 2.35E-01 |
| LS7 Score estimate |  |  |  |  |  |  |  |  |
| Adapted-LS7 score | -0.001 (-0.02-0.01) | 4.424E-01 | -0.01 (-0.02-0) | 1.57E-01 | -0.001 (-0.02-0.01) | 7.48E-01 | -0.02 (-0.03--0.01) | **1.31E-04** |

*Supplementary Table 3a presents the beta estimates from linear regression analyses examining the associations between cardiovascular-related factors (exposures) and epigenetic age acceleration (outcome).*

**Supplementary Table 3b. The association of cardiovascular-related factors with change in BA (Time 2- Time 1) as estimated by two first generation and two second generation epigenetic clocks in the AGES-RS cohort.**

| Variable | PC-Horvath |  | PC-Hannum |  | PC-PhenoAge |  | PC-GrimAge |  |
| --- | --- | --- | --- | --- | --- | --- | --- | --- |
|  | **Estimate (95% CI)** | **P-Value** | **Estimate (95% CI)** | **P-Value** | **Estimate (95% CI)** | **P-Value** | **Estimate (95% CI)** | **P-Value** |
| Smoking status |  |  |  |  |  |  |  |  |
| Never | Reference |  |  |  |  |  |  |  |
| Ex | 0.029 (-0.067 - 0.125) | 5.58E-01 | -0.021 (-0.113 - 0.071) | 6.54E-01 | 0.029 (-0.063 - 0.120) | 5.38E-01 | 0.048 (-0.048 - 0.144) | 3.29E-01 |
| Current | **0.206 (0.056 - 0.356)** | **7.04E-03** | 0.015 (-0.128 - 0.159) | 8.35E-01 | **0.150 (0.007 - 0.294)** | **3.94E-02** | -0.108 (-0.279 - 0.064) | 2.17E-01 |
| Pack-years of smoking | **0.087 (0.041 - 0.133)** | **1.90E-04** | 0.035 (-0.009 - 0.079) | 1.19E-01 | **0.067 (0.023 - 0.112)** | **2.82E-03** | **-0.169 (-0.286 - -0.052)** | **4.53E-03** |
| History of moderate to Vigorous PA |  |  |  |  |  |  |  |  |
| None | Reference |  |  |  |  |  |  |  |
| Low | -0.011 (-0.124 - 0.103) | 8.54E-01 | -0.001 (-0.110 - 0.107) | 9.81E-01 | -0.043 (-0.151 - 0.064) | 4.30E-01 | -0.066 (-0.174 - 0.042) | 2.31E-01 |
| Middle | 0.037 (-0.087 - 0.161) | 5.54E-01 | -0.009 (-0.128 - 0.109) | 8.77E-01 | -0.059 (-0.177 - 0.059) | 3.27E-01 | -0.057 (-0.176 - 0.061) | 3.42E-01 |
| High | 0.025 (-0.102 - 0.153) | 6.97E-01 | 0.026 (-0.097 - 0.148) | 6.82E-01 | -0.116 (-0.238 - 0.006) | 6.15E-02 | -0.068 (-0.190 - 0.054) | 2.76E-01 |
| PA (Continuous form) | 0.003 (-0.041 - 0.047) | 9.02E-01 | 0.007 (-0.035 - 0.049) | 7.44E-01 | -0.038 (-0.080 - 0.004) | 7.46E-02 | -0.017 (-0.059 - 0.025) | 4.34E-01 |
| BMI | -0.014 (-0.058 - 0.031) | 5.49E-01 | -0.011 (-0.053 - 0.031) | 6.14E-01 | 0.016 (-0.026 - 0.058) | 4.54E-01 | **0.057 (0.015 - 0.099)** | **7.89E-03** |
| SBP | **-0.055 (-0.100 - -0.010)** | 1.63E-02 | 0.007 (-0.037 - 0.050) | 7.67E-01 | 0.015 (-0.028 - 0.058) | 4.96E-01 | 0.013 (-0.030 - 0.056) | 5.53E-01 |
| DBP | -0.043 (-0.088 - 0.002) | 5.89E-02 | -0.021 (-0.065 - 0.022) | 3.33E-01 | 0.016 (-0.027 - 0.059) | 4.74E-01 | -0.010 (-0.054 - 0.033) | 6.33E-01 |
| Cholesterol | 0.009 (-0.045 - 0.063) | 7.37E-01 | -0.019 (-0.071 - 0.033) | 4.75E-01 | -0.007 (-0.059 - 0.044) | 7.77E-01 | **-0.056 (-0.107 - -0.004)** | **3.41E-02** |
| Glucose | -0.023 (-0.077 - 0.031) | 3.95E-01 | -0.021 (-0.072 - 0.031) | 4.31E-01 | 0.013 (-0.038 - 0.065) | 6.16E-01 | -0.013 (-0.064 - 0.039) | 6.31E-01 |
| Adapted-LS7 score | -0.003 (-0.047 - 0.041) | 8.96E-01 | -0.002 (-0.044 - 0.040) | 9.20E-01 | -0.040 (-0.082 - 0.002) | 6.24E-02 | -0.039 (-0.082 - 0.004) | 7.50E-02 |

*All* ***p-values*** *were derived from two-sided linear regression analyses. Supplementary Table 3b presents the beta estimates from linear regression analyses examining the associations between cardiovascular-related factors (exposures) and changes in BA (outcome), calculated as the difference between BA at time 2 and time 1.*
