# Supplementary material for: Mid- and late-life cardiovascular health indicators and changes in biological ageing Markers; A multi-cohort study
Source: eBioMedicine. 2025 Nov 11;122:106016. doi: 10.1016/j.ebiom.2025.106016 (PMC12657379; doi:10.1016/j.ebiom.2025.106016)
Supplement: Supplementary Table 4 [file mmc16.docx]

**Supplementary Table 4a. Description of five years follow-up change metrics for the DunedinPACE score by study, change interval and demographics**

| Mean (SD) change in DunedinPACE score by cohort and follow-up time | | | | | | | | | | | |  |
| --- | --- | --- | --- | --- | --- | --- | --- | --- | --- | --- | --- | --- |
|  | **5 years follow-up** | | | | | | | | | |  | |
| Variables | **AGES-RS** | **P** | **InCHIANTI 2013-2007** | **P** | **CARDIA Y20-Y15** | **P** | **CARDIA Y25-Y20** | **P** | **CARDIA Y30-Y25** | **P** | |  |
| N | 2,081 |  | 344 |  | 1,395 |  | 1,697 |  | 2,465 |  | |  |
| Overall mean (SD) change in DunedinPACE score | 0.037 (0.069) | - | 0.009 (0.080) | - | 0.016 (0.087) | - | 0.019 (0.079) | - | 0.017 (0.074) | - | |  |
| Sex |  |  |  |  |  |  |  |  |  |  | |  |
| M | 0.037 (0.070) | 0.824 | 0.009 (0.084) | 0.943 | 0.029 (0.081) | 1.36E-04 | 0.029 (0.072) | 3.36E-05 | 0.026 (0.069) | 1.41E-07 | |  |
| F | 0.037 (0.068) |  | 0.010 (0.077) |  | 0.010 (0.089) |  | 0.012 (0.082) |  | 0.011 (0.077) |  |  |  |
| Age category (Years)¥ | |  |  |  |  |  |  |  |  |  | |  |
| < 76 | 0.036 (0.069) | 0.539 | - | - | - | - | - | - | - | - | |  |
| >= 76 | 0.038 (0.069) |  | - | - | - | - | - | - | - | - | |  |
| Age category (Years) ¥ | |  |  |  |  |  |  |  |  |  | |  |
| 20-40 | - | - | 0.010 (0.070) | 3.97e-3 | - | - | - | - | - | - | |  |
| 40-60 | - | - | -0.018 (0.078) |  | - | - | - | - | - | - | |  |
| 60 + |  |  | 0.019 (0.082) |  | - | - | - | - | - | - | |  |
| Age category (Years) ¥ | |  |  |  |  |  |  |  |  |  | |  |
| 18 - <25 | - | - | - | - | 0.013 (0.084) | 0.058 | 0.016 (0.078) | 0.1497 | 0.018 (0.075) | 0.518 | |  |
| 25 - 30 | - | - | - | - | 0.023 (0.090) |  | 0.022 (0.080) |  | 0.016 (0.074) |  |  |  |
| Ageing shift group |  |  |  |  |  |  |  |  |  |  | |  |
| Decelerators | -0.020 (0.065) | < 2e-16 | -0.046 (0.088) | < 2e-16 | -0.053 (0.087) | < 2e-16 | -0.0392 (0.074) | < 2e-16 | -0.0411 (0.067) | < 2e-16 | |  |
| Average Agers | 0.027 (0.053) |  | 0.002 (0.066) |  | 0.009 (0.064) |  | 0.013 (0.062) |  | 0.013 (0.062) |  |  |  |
| Accelerators | 0.078 (0.071) |  | 0.061 (0.066) |  | 0.075 (0.090) |  | 0.065 (0.086) |  | 0.062 (0.073) |  |  |  |

¥Age categories were based on baseline assessments; in 2006 for AGES-RS, in 1998 for InCHIANTI and at Y0 for CARDIA.

**Supplementary Table 4b. Description of nine years follow-up change metrics for the DunedinPACE score by study, change interval and demographics**

|  | Mean (SD) change in DunedinPACE score by cohort and follow-up time | | | | |  |
| --- | --- | --- | --- | --- | --- | --- |
| 9+ years follow-up | | | | | | |
| Variables | **InCHIANTI 2007-1998** | **P** | **InCHIANTI 2013-1998** | **P** | **CARDIA Y30-Y15** | **P** |
| N | 637 |  | 364 |  | 1,394 |  |
| Overall mean (SD) change in DunedinPACE score | 0.045 (0.082) | - | 0.053 (0.091) | - | 0.047 (0.101) | - |
| Sex |  |  |  |  |  |  |
| M | 0.047 (0.084) | 0.494 | 0.058 (0.090) | 0.286 | 0.078 (0.100) | < 2e-16 |
| F | 0.043 (0.080) |  | 0.048 (0.092) |  | 0.030 (0.098) |  |
| Age category (Years) ¥ |  |  |  |  |  |  |
| 20-40 | 0.037 (0.073) | 1.26e-2 | 0.043 (0.086) | 4.35e-5 | - | - |
| 40-60 | 0.022 (0.069) |  | 0.014 (0.085) |  | - | - |
| 60 + | 0.050 (0.085) |  | 0.068 (0.091) |  | - | - |
| Age category (Years) ¥ |  |  |  |  |  |  |
| 18 - <25 | - | -  - | - | - | 0.0412 (0.101) | 1.16e-2 |
| 25 - 30 | - |  | - | - | 0.055 (0.102) |  |
| Ageing shift group |  |  |  |  |  |  |
| Decelerators | -0.028 (0.073) | < 2e-16 | 0.105 (0.079) | < 2e-16 | -0.047 (0.087) | < 2e-16 |
| Average Agers | 0.031 (0.062) |  | 0.028 (0.067) |  | 0.023 (0.073) |  |
| Accelerators | 0.095 (0.081) |  | -0.046 (0.085) |  | 0.114 (0.101) |  |

¥Age categories were based on baseline assessments; in 2006 for AGES-RS, in 1998 for InCHIANTI and at Y0 for CARDIA.

For analyses comparing two groups, **p-values** were generated from **independent t-tests**. For analyses comparing three groups, **p-values** were generated from **one-way ANOVA**. For categorical variables, **p-values** were generated using **Chi-squared tests**.
