# Supplementary material for: Mid- and late-life cardiovascular health indicators and changes in biological ageing Markers; A multi-cohort study
Source: eBioMedicine. 2025 Nov 11;122:106016. doi: 10.1016/j.ebiom.2025.106016 (PMC12657379; doi:10.1016/j.ebiom.2025.106016)
Supplement: Supplementary Table 5 [file mmc17.docx]

**Supplementary Table 5. Longitudinal associations between DunedinPACE and cardiovascular health-related risk factors and their composite score in a unified analysis of three cohorts, with and without cohort and BeadChip adjustment**

| **Variable** | **Before adjusting for BeadChip and cohort** | | | **Adjusted for BeadChip** | | | **Adjusted for both BeadChip and cohort** | | |
| --- | --- | --- | --- | --- | --- | --- | --- | --- | --- |
|  | **Beta (95% CI)** | **P** | **N** | **Beta (95% CI)** | **P** | **N** | **Beta (95% CI)** | **P** | **N** |
| **Smoking Status** |  |  |  |  |  |  |  |  |  |
| Never-smoker |  |  |  |  |  |  |  |  |  |
| Ex | 0.18 (0.15 - 0.21) | **8.72E-29** | 14,917 | 0.18 (0.15 - 0.21) | **9.82E-28** | 14,917 | 0.15 (0.12 - 0.18) | **1.17E-20** | 14,917 |
| Current | 0.43 (0.39 - 0.47) | **1.39E-97** | 14,917 | 0.42 (0.38 - 0.46) | **1.08E-95** | 14,917 | 0.39 (0.35 - 0.43) | **1.69E-83** | 14,917 |
| Pack-year of smoking | 0.39 (0.37 - 0.41) | **0.00E+00** | 15,081 | 0.41 (0.40 - 0.43) | **0.00E+00** | 15,081 | 0.41 (0.39 - 0.42) | **0.00E+00** | 15,081 |
| **History of PA** |  |  |  |  |  |  |  |  |  |
| Poor |  |  |  |  |  |  |  |  |  |
| Medium | -0.09 (-0.11 - -0.07) | **5.69E-17** | 14,937 | -0.10 (-0.12 - -0.07) | **1.21E-18** | 14,937 | -0.09 (-0.12 - -0.07) | **5.55E-18** | 14,937 |
| High | -0.14 (-0.17 - -0.12) | **9.40E-35** | 14,937 | -0.15 (-0.17 - -0.13) | **2.77E-37** | 14,937 | -0.15 (-0.17 - -0.13) | **1.04E-38** | 14,937 |
| PA-continuous | -0.11 (-0.12 - -0.10) | **2.13E-65** | 14,721 | -0.11 (-0.12 - -0.10) | **4.14E-62** | 14,721 | -0.09 (-0.10 - -0.07) | **8.63E-36** | 14,721 |
| **Anthropometry** |  |  |  |  |  |  |  |  |  |
| BMI | 0.21 (0.19 - 0.22) | **3.58E-157** | 15,009 | 0.21 (0.20 - 0.23) | **1.00E-163** | 15,009 | 0.25 (0.24 - 0.27) | **1.82E-231** | 15,009 |
| **Biometrics** |  |  |  |  |  |  |  |  |  |
| SBP | 0.03 (0.02 - 0.04) | **5.01E-09** | 14,992 | 0.03 (0.02 - 0.04) | **5.00E-09** | 14,992 | 0.02 (0.01 - 0.04) | **1.34E-05** | 14,992 |
| DBP | 0.02 (0.01 - 0.03) | **1.40E-05** | 14,989 | 0.02 (0.01 - 0.03) | **1.41E-04** | 14,989 | 0.02 (0.01 - 0.03) | **3.03E-06** | 14,989 |
| Cholesterol | -0.09 (-0.11 - -0.08) | **4.57E-58** | 15,058 | -0.09 (-0.10 - -0.08) | **1.68E-53** | 15,058 | -0.07 (-0.08 - -0.06) | **1.40E-34** | 15,058 |
| Fasting blood glucose | 0.06 (0.05 - 0.07) | **6.83E-27** | 14,623 | 0.06 (0.05 - 0.07) | **2.86E-26** | 14,623 | 0.05 (0.04 - 0.07) | **8.27E-21** | 14,623 |
| **LS7 score estimate** |  |  |  |  |  |  |  |  |  |
| Adapted-LS7 score | -0.15 (-0.16 - -0.14) | **1.34E-111** | 13,709 | -0.15 (-0.16 - -0.13) | **1.44E-106** | 13,709 | -0.14 (-0.15 - -0.13) | **1.73E-96** | 13,709 |

All ***p-values*** were derived from two-sided linear mixed model regression analyses. DNA methylation was assayed using the Illumina 450k array in the InCHIANTI cohort and the MethylEPIC array in the AGES-RS and CARDIA cohorts. Variables from the three cohorts were merged into a single long-format dataset, and longitudinal associations between cardiovascular health–related factors and DunedinPACE were assessed using linear mixed-effects models. All models were adjusted for sex, chronological age, white blood cell composition, BeadChip and cohort.

N = number of repeated observations, exceed the number of unique participants.
